# A superior colliculus-originating circuit prevents cocaine reinstatement via VR-based eye movement desensitization treatment

**DOI:** 10.1093/nsr/nwae467

**Published:** 2024-12-26

**Authors:** Yang Liu, Zi-Xiang Zhou, Qiu Lv, Guan Huang, Han Zhang, Ye-Qin Wang, Jian-Guo Chen, Fang Wang

**Affiliations:** State Key Laboratory for Diagnosis and Treatment of Severe Zoonotic Infectious Diseases, Department of Pharmacology, School of Basic Medicine, Tongji Medical College, Huazhong University of Science and Technology, Wuhan 430030, China; State Key Laboratory for Diagnosis and Treatment of Severe Zoonotic Infectious Diseases, Department of Pharmacology, School of Basic Medicine, Tongji Medical College, Huazhong University of Science and Technology, Wuhan 430030, China; State Key Laboratory for Diagnosis and Treatment of Severe Zoonotic Infectious Diseases, Department of Pharmacology, School of Basic Medicine, Tongji Medical College, Huazhong University of Science and Technology, Wuhan 430030, China; State Key Laboratory for Diagnosis and Treatment of Severe Zoonotic Infectious Diseases, Department of Pharmacology, School of Basic Medicine, Tongji Medical College, Huazhong University of Science and Technology, Wuhan 430030, China; State Key Laboratory for Diagnosis and Treatment of Severe Zoonotic Infectious Diseases, Department of Pharmacology, School of Basic Medicine, Tongji Medical College, Huazhong University of Science and Technology, Wuhan 430030, China; State Key Laboratory for Diagnosis and Treatment of Severe Zoonotic Infectious Diseases, Department of Pharmacology, School of Basic Medicine, Tongji Medical College, Huazhong University of Science and Technology, Wuhan 430030, China; State Key Laboratory for Diagnosis and Treatment of Severe Zoonotic Infectious Diseases, Department of Pharmacology, School of Basic Medicine, Tongji Medical College, Huazhong University of Science and Technology, Wuhan 430030, China; The Key Laboratory for Drug Target Researches and Pharmacodynamic Evaluation of Hubei Province, Wuhan 430030, China; The Research Center for Depression, Tongji Medical College, Huazhong University of Science and Technology, Wuhan 430030, China; Hubei Shizhen Laboratory, Wuhan 430030, China; State Key Laboratory for Diagnosis and Treatment of Severe Zoonotic Infectious Diseases, Department of Pharmacology, School of Basic Medicine, Tongji Medical College, Huazhong University of Science and Technology, Wuhan 430030, China; The Key Laboratory for Drug Target Researches and Pharmacodynamic Evaluation of Hubei Province, Wuhan 430030, China; The Research Center for Depression, Tongji Medical College, Huazhong University of Science and Technology, Wuhan 430030, China; Hubei Shizhen Laboratory, Wuhan 430030, China

**Keywords:** VR treatment, cocaine, reinstatement, SCi^CaMKIIα^→LC^TH^→dCA3 circuit, environmental cues, dopamine

## Abstract

While Virtual Reality (VR) technology shows promise in the management of substance use disorders, the development of an effective VR-based extinction procedure remains lacking. In this study, we developed a VR-based eye movement desensitization and reprocessing extinction training program tailored for mice. We found that this VR treatment during cocaine extinction prevents reinstatement by suppressing the hyperactivation of glutamatergic excitatory neurons in the intermediate layers of the superior colliculus (SCi^CaMKIIα^) during exposure to environmental cues. Additionally, SCi^CaMKIIα^ neurons innervate tyrosine hydroxylase-positive neurons in the locus coeruleus (LC^TH^). Environmental cues trigger stronger phasic activation of LC^TH^ neurons through this SCi^CaMKIIα^→LC^TH^ projection, leading to increased dopamine release onto the dorsal CA3 (dCA3) region, thereby facilitating reinstatement. Furthermore, we demonstrate that VR treatment effectively inhibits the neural circuitry involving SCi^CaMKIIα^→LC^TH^→dCA3 in response to environmental cues, thus preventing cocaine reinstatement. Our findings suggest that VR treatment may represent a promising strategy for achieving drug abstinence.

## INTRODUCTION

The management of substance use disorders remains a significant challenge [1], particularly in the case of cocaine use disorder, which is characterized by a high relapse rate and imposes a substantial global burden [2]. Relapse remains the primary challenge in managing cocaine dependence [3], largely driven by environmental drug-related cues that trigger cravings and relapse behaviors [4]. Rodent studies have similarly demonstrated that environmental drug-related cues induce reinstatement of drug-seeking behavior, highlighting a shared relapse mechanism across species [5]. However, interventions aimed at mitigating cue-induced relapse, such as extinction training, have shown limited efficacy, largely due to their reduced ecological validity, particularly the absence of contextual and complex cues in experimental designs [6,7].

Recently, Virtual Reality (VR) has gained attention for its potential application in managing substance use disorders [8], emerging as a promising tool to address these challenges. Characterized by its ability to create immersive three-dimensional environments closely resembling real-world settings, VR allows individuals to fully interact with their environment, generating immersive feelings and experiences [9]. VR has been demonstrated to augment the efficacy of conventional cue-exposure treatment for alcohol use disorder through immersive real-life simulations [10]. Studies have also indicated that VR-based motivational reinforcement combined with desensitization interventions reduces cravings for methamphetamine to a certain extent [11]. However, the application of VR technology in managing cocaine use disorder remains limited by a lack of neurobiological grounding and the absence of well-established, effective strategies.

The alternating bilateral sensory stimulation (ABS) [12], a technique based on eye movement desensitization and reprocessing, has been widely utilized for the treatment of post-traumatic stress disorder [13]. As a visual stimulation-based treatment to facilitate memory extinction, ABS holds promise for enhancing its effectiveness through integration with VR technology. Research suggests that the superior colliculus (SC), a critical brain region for integrating visual information [14], serves as a key site for ABS treatment [13]. The SC is recognized for regulating behavioral responses to visual cues [15], particularly cue-reward associations [16], indicating its potential modulation by reward-related visual cues. Remarkably, SC neuron excitability increases following cocaine exposure [17], yet our comprehension of the neural mechanisms underlying SC functions in substance use disorders remains ambiguous. Studies indicate that SC neurons project to tyrosine hydroxylase-positive neurons in the locus coeruleus (LC^TH^) [18], crucial for attention regulation [19] and activation by reward-related cues [20,21]. Furthermore, LC^TH^ neurons contribute to the formation and retrieval of spatial-contextual memory through their projections to the dorsal hippocampus (dHip) [22,23], a region crucial for context-induced cocaine seeking in rats [24]. These findings highlight the role of the SC in mediating visual cues and triggering reinstatement at the level of neural circuits. However, further research is required to determine whether the SC and its downstream pathways are also involved in the mediation of extinction.

In this study, we established a mouse model of cocaine-induced conditioned place preference (CPP) to assess the effectiveness of different extinction strategies in preventing cue-induced reinstatement. Among the approaches tested, VR-based alternating bilateral sensory stimulation (VR-ABS) demonstrated the greatest efficacy in preventing cocaine reinstatement. To explore the underlying mechanism of VR-ABS in mitigating reinstatement, we identified the intermediate layers of the superior colliculus (SCi^CaMKIIα^) as a key target by utilizing c-Fos as a neuronal activity marker. Additionally, patch-clamp recordings revealed that VR-ABS treatment reversed the increased excitability of SCi^CaMKIIα^ neurons in cocaine-CPP-trained mice. To further elucidate the neural circuitry involved in the effects of VR-ABS on reinstatement, we employed *in vivo* fiber photometry, multi-channel electrophysiological recording, chemogenetics, and optogenetics. Our findings uncovered a circuit originating from SCi^CaMKIIα^ neurons projecting to tyrosine hydroxylase-positive neurons in the locus coeruleus (LC^TH^) and extending to the dorsal CA3 (dCA3) region of the hippocampus. VR-ABS treatment was shown to reduce the selective hyperactivation of the SCi^CaMKIIα^→LC^TH^→dCA3 circuit triggered by environmental cues, thereby preventing cocaine reinstatement. These findings highlight the potential of VR-based strategies as a promising avenue for managing substance use disorders.

## RESULTS

### Manipulating environmental visual cues using VR effectively induces cocaine reinstatement

**Figure 1.**
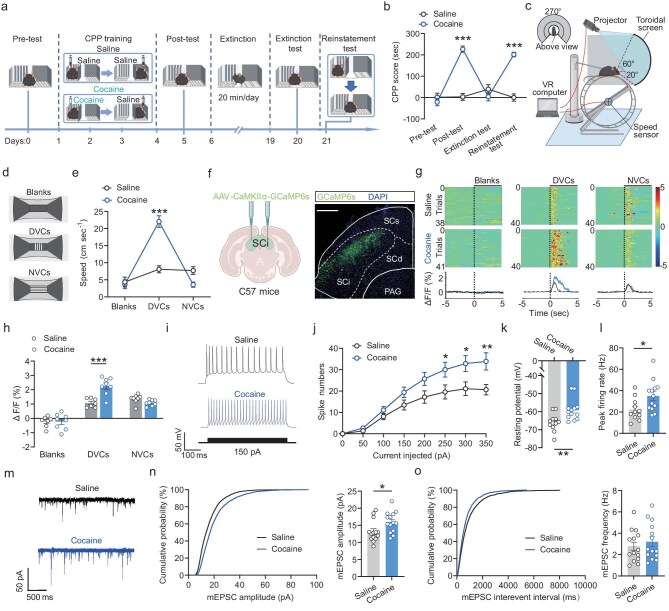
Enhanced activation of SCi^CaMKIIα^ neurons during cocaine reinstatement induced by environmental cues. (a) Schematic of the experimental timeline for cocaine-induced CPP training, extinction, and cocaine reinstatement testing. (b) Mice exhibited increased CPP scores following CPP training, which decreased after extinction, but were subsequently elevated again after cocaine reinstatement. (c and d) Experimental arrangement (c) and visual stimulation scheme (d) for detecting cocaine reinstatement induced by environmental cues. (e) Increased locomotion velocity observed in cocaine-CPP-trained mice following DVC presentation manipulated by the VR system. (f) Left: Schematic depiction of AAV injection into SCi. Right: Representative images showing GCaMP6s expression. Scale bar: 500 μm. (g) Heatmaps and average calcium transients illustrating enhanced responses to DVCs in the VR environment in cocaine-CPP-trained mice. Shaded areas represent error bars. Color scales on the right indicate Δ*F/F* values. (h) Increased peak calcium transients in response to DVCs in the VR environment in cocaine-CPP-trained mice. (i and j) Representative AP traces (i) and spike counts (j) demonstrating elevated firing rates of SCi^CaMKIIα^ neurons in cocaine-CPP-trained mice. (k and l) Enhanced membrane depolarization (k) and increased peak firing rate (l) of SCi^CaMKIIα^ neurons in cocaine-CPP-trained mice. (m) Representative traces recorded from SCi^CaMKIIα^ neurons. (n and o) Enhanced amplitudes (n) of mEPSCs were observed in SCi^CaMKIIα^ neurons from cocaine-CPP-trained mice; however, frequencies (o) remained unchanged. SCs, superficial layers of the superior colliculus; SCi, intermediate layers of the superior colliculus; SCd, deep layers of the superior colliculus; PAG, periaqueductal gray. All data are presented as mean ± s.e.m.; statistical significance is denoted as *p < 0.05, **p < 0.01, or ***p < 0.001.

Initially, we used C57BL/6 (C57) mice to establish a cocaine-induced conditioned place preference (CPP) model (Fig. 1a). Since cocaine has biphasic effects on both CPP and locomotor activity, we tested different doses to assess their impact. Notably, a 10 mg/kg dose significantly induced place preference without the marked increase in locomotor activity observed with 20 mg/kg (Fig. S1a, b), making it more suitable for reinstatement studies. Therefore, we selected 10 mg/kg for subsequent experiments. After 4 days of CPP training, mice developed strong place preference, and 14 days of extinction training effectively reduced CPP scores. Re-exposure to environmental cues reinstated place preference, inducing cocaine reinstatement (Fig. 1b). No significant sex differences were observed (Fig. S1c), therefore equal numbers of male and female mice were included in later experiments.

Next, we investigated the role of various sensory cues provided within the cocaine-paired chamber of the CPP apparatus in inducing cocaine reinstatement. The chamber paired with cocaine contained two distinct sensory conditioned cues: visual cues consisting of white walls with black vertical stripes and tactile cues featuring a metal grid floor. For testing, CPP-trained mice were re-exposed to the CPP apparatus, either with visual cues only or tactile cues alone. Our findings revealed that visual cues, rather than tactile cues, significantly triggered cocaine reinstatement (Fig. S1d, e). Moreover, when the visual input of cocaine-CPP-trained mice was obstructed using small blindfolds, re-exposure to the environmental cues failed to induce cocaine reinstatement (Fig. S1f, g). These results underscore the critical role of environmental visual cues in triggering cocaine reinstatement.

To further investigate the specificity of visual cues, we replaced the drug-related visual cues (DVCs) in the cocaine-paired chamber with non-drug-related visual cues (NVCs) characterized by white walls with black horizontal stripes. Re-exposure of cocaine-CPP-trained mice to DVCs successfully elicited cocaine reinstatement, whereas exposure to NVCs failed to produce a significant increase in CPP scores (Fig. S1h, i). Interestingly, even localized re-exposure to DVCs in the environment was sufficient to trigger cocaine reinstatement (Fig. S1j, k). These findings emphasize the potent and specific role of DVCs in facilitating cocaine reinstatement within environmental contexts.

To further investigate how cocaine reinstatement is triggered by specific environmental cues, we employed VR technology, which utilizes primarily visual sensory stimuli, in a rodent model of cocaine use disorder. Based on previous literature [25], we constructed a visual VR system for head-restrained mice, as depicted in Fig. 1c. This system was designed to create a virtual CPP testing environment, allowing us to precisely manipulate the visual cues. We recorded the conditioned locomotor activity of mice in response to visual cues in the VR environment to evaluate their reactions to these cues (Fig. 1d; Movie S1). Since increased locomotor activity in response to drug-related cues reflects the preference of animals, this approach served as an indirect measure of place preference behavior within the virtual environment. Our results showed that cocaine-CPP-trained mice exhibited significantly increased conditioned locomotor activity in response to DVC stimuli in the VR environment compared to the control group (Fig. 1e). In contrast, no significant increase in speed was observed in response to NVCs. These findings demonstrate that manipulating environmental visual cues using VR technology can reliably induce and assess cocaine reinstatement in mice.

### The activity of SCi^CaMKIIα^ neurons in response to environmental cues is heightened in cocaine-CPP-trained mice

To investigate the responsiveness of SC neurons to visual cues in a VR environment, we assessed the expression of the immediate early gene *c-Fos* in both control and cocaine-CPP-trained mice. Robust c-Fos expression was observed in the SC intermediate (SCi) region of cocaine-CPP-trained mice when exposed to DVCs in the VR environment (Fig. S2a, b). Most c-Fos-expressing neurons in the SCi exhibited colocalization with Ca^2+^/calmodulin-dependent protein kinase IIα (CaMKIIα) and vesicular glutamate transporter 1 (VGLUT1) antibodies (Fig. S2c, d), indicating that glutamatergic excitatory (SCi^CaMKIIα^) neurons primarily mediate the response to DVCs.

Subsequently, fiber photometry was utilized to investigate the real-time activity of SCi^CaMKIIα^ neurons in response to visual cues in the VR environment (Fig. 1f). The fluorescence intensity of SCi^CaMKIIα^ neurons expressing GCaMP6s was significantly higher in cocaine-CPP-trained mice exposed to DVCs in the VR environment (Fig. 1g, h). Additionally, employing *in vivo* multi-tetrode recording, we detected an elevated firing rate of SCi neurons in cocaine-CPP-trained mice upon exposure to DVCs in the VR environment (Fig. S3a, b). These results suggest an augmented activation of SCi^CaMKIIα^ neurons in response to DVC stimuli in the VR environment in mice trained with cocaine-CPP.

Subsequently, we explored the neural mechanisms underlying the increased activity of SCi^CaMKIIα^ neurons in response to DVCs. Whole-cell patch-clamp recordings were utilized to assess the impact of cocaine-CPP training on the excitability of SCi^CaMKIIα^ neurons. Our findings revealed that step depolarizing current injections induced an increased number of spikes in SCi^CaMKIIα^ neurons of cocaine-CPP-trained mice (Fig. 1i and j). Furthermore, compared to control mice, enhanced membrane depolarization and an increased firing rate of action potentials (APs) were observed in SCi^CaMKIIα^ neurons from cocaine-CPP-trained mice (Fig. 1k and l). Additionally, cocaine-CPP training led to amplified amplitudes of miniature excitatory postsynaptic currents (mEPSCs) in SCi^CaMKIIα^ neurons (Fig. 1m–o). These results suggest that cocaine-CPP training enhances the responsiveness of SCi^CaMKIIα^ neurons to environmental cues, potentially by increasing neuronal excitability.

To investigate the role of increased excitability in SCi^CaMKIIα^ neurons in environmental cue-induced cocaine reinstatement, we selectively suppressed SCi^CaMKIIα^ neuron activity by expressing the inhibitory DREADD receptor (hM4Di) for chemogenetic manipulation (Fig. S4a, b). Administration of clozapine N-oxide dihydrochloride (CNO) effectively suppressed the excitability of SCi^CaMKIIα^ neurons, thereby inhibiting cocaine reinstatement triggered by re-exposure to DVCs (Fig. S4c). Additionally, chemogenetic inhibition of SCi^CaMKIIα^ neuron excitability in cocaine-CPP-trained mice resulted in a significant reduction in the velocity of the mice towards DVCs in the VR environment (Fig. S4d). These findings highlight the critical role of SCi^CaMKIIα^ neuron excitability in cocaine reinstatement triggered by environmental cues.

### VR-ABS treatment prevents cocaine reinstatement by reducing the heightened excitability of SCi^CaMKIIα^ neurons in cocaine-CPP-trained mice

**Figure 2.**
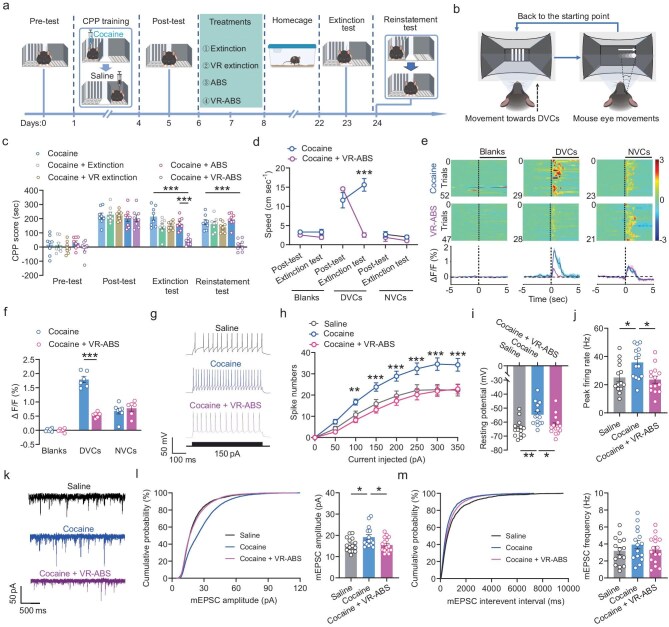
VR-ABS prevents environmental cue-induced cocaine reinstatement by reducing the excitability of SCi^CaMKIIα^ neurons. (a) Experimental schematic illustrating various extinction strategies applied to cocaine-CPP-trained mice. (b) Schematic diagram of the VR-ABS experimental setup. Mice encounter DVC stimuli at the far end of the virtual CPP apparatus via a VR device. As mice approach the visual cue, DVCs transition into a slow-moving white dot, eliciting eye movements. The dot vanishes upon reaching the edge, and mice are returned to the starting point. (c) VR-ABS significantly reduced CPP scores in cocaine-CPP-trained mice and persistently suppressed cocaine reinstatement triggered by environmental cues. (d) VR-ABS decreased locomotor velocity during reinstatement testing in cocaine-CPP-trained mice. (e) Heatmaps and average calcium transients demonstrate that VR-ABS treatment significantly decreased the hyperactivation of SCi^CaMKIIα^ neurons induced by DVCs in the VR environment in cocaine-CPP-trained mice. Shaded areas represent error bars. Color scales on the right indicate Δ*F/F* values. (f) Increased peak calcium transients of SCi^CaMKIIα^ neurons induced by DVCs in cocaine-CPP-trained mice were attenuated following VR-ABS treatment. (g and h) Representative AP traces (g) and spike counts (h) showing the reversal of increased firing rates of SCi^CaMKIIα^ neurons in cocaine-CPP-trained mice by VR-ABS. (i and j) VR-ABS reversed the increased membrane depolarization (i) and heightened peak firing rate (j) of SCi^CaMKIIα^ neurons in cocaine-CPP-trained mice. (k) Representative traces recorded from SCi^CaMKIIα^ neurons. (l and m) VR-ABS reversed the enhanced amplitudes (l) of mEPSCs observed in SCi^CaMKIIα^ neurons of cocaine-CPP-trained mice; however, it had no effect on frequencies (m). All data are presented as mean ± s.e.m.; statistical significance is indicated as *p < 0.05, **p < 0.01, or ***p < 0.001.

Previous studies have suggested that VR technology may more effectively elicit cocaine cravings and drug-seeking behaviors [8]. We further investigated whether the immersive experience provided by VR technology enhances the efficacy of existing extinction training in suppressing cocaine reinstatement. We first trained C57 mice using a cocaine-induced CPP model. Following this, we implemented various extinction training protocols over three consecutive days, including traditional extinction training, ABS training, VR-based extinction training, and VR-ABS training (Fig. 2a; Movie S2). Among these approaches, VR-ABS proved most effective in preventing environmental cue-induced cocaine reinstatement (Fig. 2b, c). Additionally, this treatment also sustained the mitigation of the increase in locomotor speed induced by DVCs in the VR environment (Fig. 2d). These findings highlight the potential of VR-ABS treatment as an effective strategy for managing substance use disorders.

Subsequently, we further investigated the impact of VR-ABS treatment on the functionality of SCi^CaMKIIα^ neurons. Using *in vivo* fiber photometry measurements, we found that VR-ABS treatment reversed the heightened fluorescence intensity of GCaMP6s-expressing SCi^CaMKIIα^ neurons in cocaine-CPP-trained mice in response to DVC stimulation (Fig. 2e, f). Additionally, whole-cell patch-clamp recording was employed to assess the impact of VR-ABS treatment on the excitability of SCi^CaMKIIα^ neurons. Our findings revealed that VR-ABS treatment reversed the increased spike activity induced by step depolarizing current injections in SCi^CaMKIIα^ neurons of cocaine-CPP-trained mice (Fig. 2g, h). Furthermore, VR-ABS treatment reversed the enhanced membrane depolarization and the elevated firing rate of APs observed in SCi^CaMKIIα^ neurons from cocaine-CPP-trained mice (Fig. 2i, j). Furthermore, this treatment reduced the amplitudes of mEPSCs of SCi^CaMKIIα^ neurons in cocaine-CPP-trained mice (Fig. 2k–m). Overall, these findings suggest that VR-ABS treatment reduces cocaine reinstatement by decreasing the hyperactivity of SCi^CaMKIIα^ neurons in cocaine-CPP-trained mice.

### Modulation of the SCi^CaMKIIα^→LC pathway modulates cocaine reinstatement triggered by environmental cues

**Figure 3.**
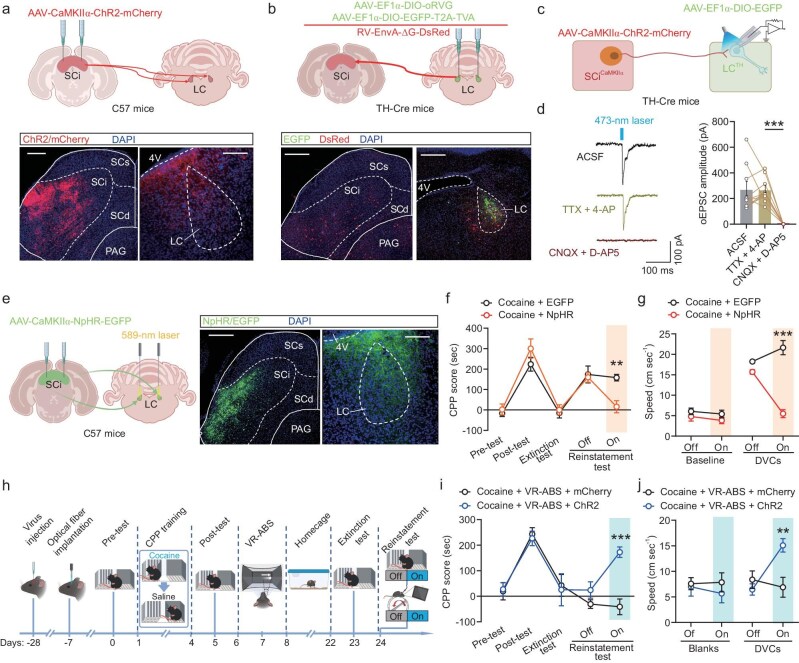
The SCi^CaMKIIα^→LC circuit mediates cocaine reinstatement triggered by environmental cues. (a) Top: Schematic depicting AAV injection. Bottom: Representative immunofluorescence images displaying ChR2-mCherry expression in SCi^CaMKIIα^ neurons and LC terminals. Scale bars: SCi, 500 μm; LC, 100 μm. (b) Top: Viral injection for retrograde tracing in TH-Cre mice. Bottom: Representative images showing the expression of RV-DsRed and EGFP fluorescence. Scale bars: LC, 200 µm; SCi, 500 µm. (c) Schematic illustrating whole-cell patch-clamp recording of LC^TH^ neurons during optogenetic stimulation of SCi^CaMKIIα^ projections. (d) oEPSCs were confirmed as monosynaptic by persisting after axon terminal stimulation of SCi^CaMKIIα^ populations with TTX + 4-AP. They were significantly reduced by NMDA and AMPA receptor blockade with D-AP5 and CNQX. (e) Left: AAV injection in the SCi with LC optical fiber implantation. Right: Representative images of NpHR-EGFP expression in SCi^CaMKIIα^ neurons and their terminals in LC. Scale bars: SCi, 500 μm; LC, 100 μm. (f and g) Optogenetic inhibition of SCi^CaMKIIα^→LC projections reduced CPP scores during the reinstatement test (g) and decreased locomotor velocity during DVC stimulation (f) in cocaine-CPP-trained mice. (h) Experimental timeline schematic for optogenetic activation detection after VR-ABS treatment. (i and j) Optogenetic activation of SCi^CaMKIIα^→LC projections reversed the VR-ABS–induced reduction in CPP scores during the reinstatement test (i), and also increased locomotor velocity during DVC stimulation (j) in cocaine-CPP-trained mice. SCs, superficial layers of the superior colliculus; SCi, intermediate layers of the superior colliculus; SCd, deep layers of the superior colliculus; PAG, periaqueductal gray; 4V, fourth ventricle. All data are presented as mean ± s.e.m.; statistical significance is indicated as **p < 0.01, or ***p < 0.001.

To further elucidate the neurocircuitry mechanism by which VR-ABS treatment reduces cocaine reinstatement via SCi^CaMKIIα^ neurons, an adeno-associated virus (AAV) containing the CaMKIIα promoter was injected into the SCi of C57 mice. As shown in Fig. 3a, dense SCi^CaMKIIα^ projection fibers and terminals labeled with mCherry were identified in the LC region, which regulates attention to visual cues [20,21,26]. Considering that LC is a major hub of tyrosine hydroxylase (LC^TH^) neurons in the brain [27], a monosynaptic tracing approach mediated by the rabies virus (RV) was used to label neurons directly presynaptic to targeted LC^TH^ neurons in TH-Cre mice. To achieve this, we initially injected Cre-dependent helper viruses (a combination of AAV-EF1α-DIO-EGFP-T2A-TVA and AAV-EF1α-DIO-oRVG) into the LC. After a period of 3 weeks, the RV-EnvA-ΔG-DsRed was injected into the same site to retrogradely infect projection neurons, including those originating in the SCi (Fig. 3b).

To validate the excitatory currents induced by SCi^CaMKIIα^ projections in LC^TH^ neurons, we performed whole-cell patch-clamp recordings on LC^TH^ neurons derived from TH-Cre mice, which were injected with a Cre-dependent viral vector expressing EGFP in the LC, along with a viral vector expressing channelrhodopsin-2 (ChR2) tagged with mCherry under the control of the CaMKIIα promoter in the SCi (Fig. 3c). The optogenetically-evoked excitatory postsynaptic currents (oEPSCs) observed in LC^TH^ neurons were elicited by stimulating axon terminals expressing ChR2 originating from the SCi^CaMKIIα^ population. These oEPSCs could be effectively suppressed by antagonizing α-amino-3-hydroxy-5-methyl-4-isoxazolepropionic acid (AMPA) and N-methyl-D-aspartate (NMDA) glutamate receptors, indicating that SCi^CaMKIIα^ neurons projecting to LC^TH^ represent monosynaptic glutamatergic projections (Fig. 3d). These findings substantiate the presence of the SCi^CaMKIIα^→LC^TH^ circuit, where SCi^CaMKIIα^ neurons innervate LC^TH^ neurons through monosynaptic glutamatergic projections.

To investigate whether SCi→LC^TH^ projection neurons are activated by environmental cues, we employed a Cre-dependent retrograde RV tracing strategy to label neurons projecting to LC^TH^ neurons with DsRed. We found that a substantial proportion, ∼63.1%, of these neurons exhibited co-localization with c-Fos activated by DVC stimulation in the VR environment (Fig. S5a, b), indicating that the majority of SCi→LC^TH^ projection neurons are activated by DVCs in cocaine-CPP-trained mice. Additionally, fiber photometry was used to monitor the real-time activity of SCi→LC projection neurons in response to visual cues. Initially, a retrograde AAV viral strategy was employed to selectively express GCaMP6s in SCi→LC projection neurons (Fig. S5c). We observed that DVCs induced a robust increase in the fluorescence intensity of GCaMP6s-expressing SCi→LC projection neurons in cocaine-CPP-trained mice in the VR environment (Fig. S5d, e), indicating enhanced activation of these neurons in response to environmental cues.

To further explore the role of SCi→LC projection neurons in environmental cue-induced cocaine reinstatement, we utilized a retrograde AAV viral approach to selectively express hM4Di in SCi→LC projection neurons (Fig. S5f). Chemogenetic suppression of SCi→LC projection neuron activity via CNO treatment in cocaine-CPP-trained mice reduced cocaine reinstatement triggered by re-exposure to DVCs (Fig. S5g). This suppression also decreased the velocity of mice when approaching DVCs in the VR environment (Fig. S5h). These results suggest that SCi→LC projection neurons play a key role in mediating cocaine reinstatement.

To rule out the possibility that the results were influenced by the inhibition of collateral projections to other brain areas, we conducted an optogenetic experiment aimed at suppressing the activity of SCi^CaMKIIα^ projection terminals in the LC. Specifically, a viral vector was utilized to express the inhibitory opsin NpHR under the control of the CaMKIIα promoter in SCi^CaMKIIα^→LC projection terminals, which were then illuminated with a yellow laser (589 nm) during behavior testing (Fig. 3e). This manipulation resulted in a reduction of CPP scores in cocaine-CPP-trained mice during the reinstatement phase (Fig. 3f). Furthermore, optogenetic inhibition of the SCi^CaMKIIα^→LC projection decreased the velocity of cocaine-CPP-trained mice when approaching DVCs in the VR environment (Fig. 3g), suggesting a direct link between SCi^CaMKIIα^→LC projection activity and cocaine reinstatement triggered by environmental cues.

To determine whether the SCi^CaMKIIα^→LC projection mediates the inhibitory effect of VR-ABS on environmental cue-induced cocaine reinstatement, we optogenetically activated the SCi^CaMKIIα^ projection expressing ChR2 by delivering 473-nm laser illumination to the LC (Fig. 3h). Our results demonstrated that optogenetic activation of the SCi^CaMKIIα^→LC projection during the reinstatement phase reversed the VR-ABS–induced suppression of cocaine reinstatement (Fig. 3i). Additionally, optogenetic activation of this projection increased locomotor velocity in response to DVCs in the VR environment in VR-ABS–treated mice (Fig. 3j). These findings suggest that activation of the SCi^CaMKIIα^→LC projection facilitates cocaine reinstatement, while VR-ABS prevents cocaine reinstatement induced by environmental cues by inhibiting this projection.

### LC^TH^ neurons exhibit enhanced phasic activity in response to environmental cues in cocaine-CPP-trained mice

**Figure 4.**
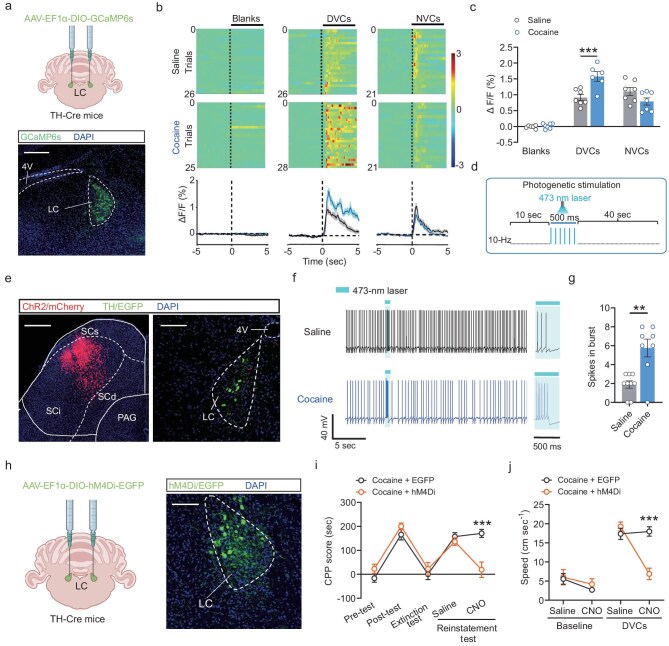
Enhanced phasic activation of LC^TH^ neurons during cocaine reinstatement induced by environmental cues. (a) Top: Schematic diagram illustrating virus injection. Bottom: Representative images displaying GCaMP6s expression in LC^TH^ neurons. Scale bar: 200 μm. (b) Heatmaps and average calcium transients depict increased calcium transients of LC^TH^ neurons in response to DVCs in the VR environment. Shaded areas represent error bars. Color scales on the right indicate Δ*F/F* values. (c) Peak calcium transients increased in response to DVC in the VR environment in mice trained with cocaine-CPP. (d) Whole-cell patch recording of LC^TH^ neurons during optogenetic stimulation (473 nm, 5 mW, 10 Hz, 10-ms pulse) of SCi^CaMKIIα^ projections. (e) Representative images illustrating AAV injections in the SCi and LC regions of TH-Cre mice to express ChR2 fused with mCherry in SCi^CaMKIIα^ neurons and EGFP in LC^TH^ neurons. Scale bars: SCi, 500 μm; LC, 100 μm. (f and g) Representative AP traces (f) and spike counts in bursts (g) demonstrate an increased number of APs during phasic firing in LC^TH^ neurons induced by optogenetic activation of SCi^CaMKIIα^ projections in cocaine-CPP-trained mice. (h) Schematic (left) and representative immunofluorescence images (right) showing hM4Di-mCherry expression in LC^TH^ neurons. Scale bars: 100 µm. (i and j) Chemogenetic silencing of LC^TH^ neurons reduced CPP scores during the reinstatement test (i) and decreased locomotor velocity in response to DVC stimulation (j) in cocaine-CPP-trained mice. 4V, fourth ventricle. All data are presented as mean ± s.e.m.; statistical significance is indicated as **p < 0.01, or ***p < 0.001.

To investigate the responsiveness of LC^TH^ neurons to environmental cues, *in vivo* fiber photometry recordings were conducted specifically targeting LC^TH^ neurons (Fig. 4a). The fluorescence intensity of GCaMP6s-expressing LC^TH^ neurons in cocaine-CPP-trained mice was significantly elevated in response to DVCs during the reinstatement phase compared to control mice (Fig. 4b, c). This result indicates heightened activation of LC^TH^ neurons in mice trained with cocaine-CPP.

Previous studies have demonstrated that phasic activation of LC^TH^ neurons correlates with behavioral responses to salient stimuli [28]. To investigate the mechanisms by which LC^TH^ neurons regulate cocaine reinstatement triggered by visual cues, we performed *in vivo* multi-tetrode recordings to monitor the real-time electrical activity of LC neurons. Our findings showed that the phasic firing of LC neurons in response to DVCs in the VR environment was significantly enhanced during the reinstatement phase in cocaine-CPP-trained mice compared to control mice (Fig. S6a–e). Subsequently, we employed a combined approach involving optogenetics and electrophysiological recordings to investigate the induction of phasic firing in LC^TH^ neurons by SCi^CaMKIIα^ neuronal input (Fig. 4d). TH-Cre mice were injected with a Cre-dependent viral vector expressing EGFP in the LC, along with a viral vector expressing ChR2 tagged with mCherry under the regulation of the CaMKIIα promoter in the SCi (Fig. 4e). We measured the action potentials of LC^TH^ neurons while stimulating ChR2-expressing terminals of SCi^CaMKIIα^ neurons. Notably, we observed that optogenetic stimulation of SCi^CaMKIIα^ neuronal input induced stronger phasic firing in LC^TH^ neurons in cocaine-CPP-trained mice compared to control mice (Fig. 4f, g). These results suggest that exposure to environmental cues enhances the phasic activation of LC^TH^ neurons via the SCi^CaMKIIα^→LC^TH^ projection, thereby facilitating cocaine reinstatement in mice.

To further investigate the regulatory role of LC^TH^ neurons downstream of SCi^CaMKIIα^ projections in cocaine reinstatement, we employed chemogenetic inhibition of LC^TH^ neurons (Fig. 4h). Suppression of LC^TH^ neuronal activity led to a reduction in CPP scores in cocaine-CPP-trained mice during the reinstatement phase (Fig. 4i). Additionally, this manipulation decreased the locomotor velocity in response to DVCs in the VR environment (Fig. 4j). These findings highlight the crucial role of LC^TH^ neuron activation in regulating environmental cue-induced cocaine reinstatement.

### Phasic activation of LC^TH^→dCA3 circuit is essential for environmental cue-induced cocaine reinstatement

**Figure 5.**
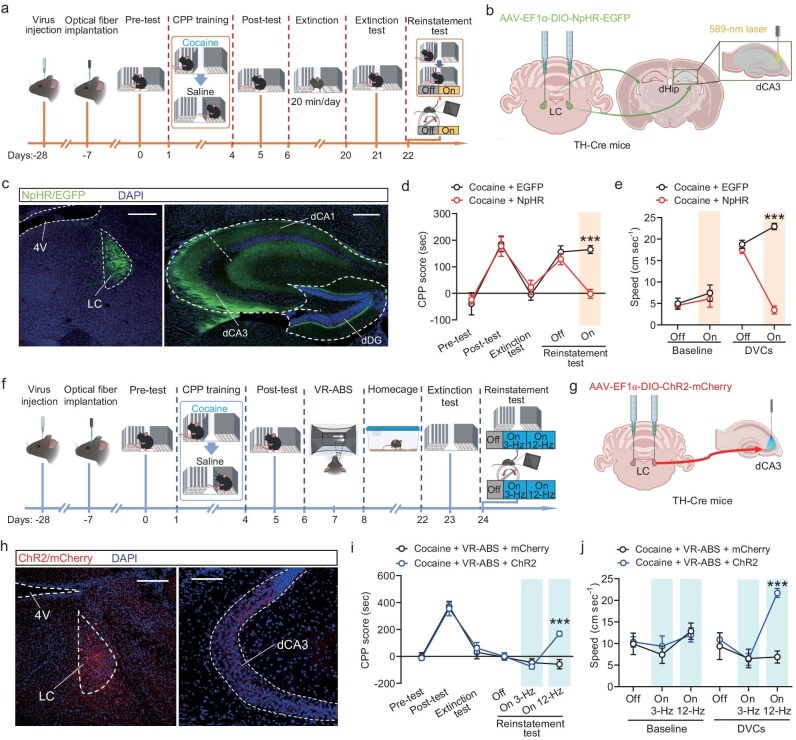
Phasic activation of LC^TH^→dCA3 circuits mediates cocaine reinstatement induced by environmental cues. (a) Experimental timeline schematic. (b) TH-Cre mice received AAV injections in the LC and underwent optical fiber implantation above the dCA3. (c) Representative immunofluorescence images displaying the expression of NpHR-EGFP in LC^TH^ neurons and their terminals in the dCA3. Scale bars: LC, 200 μm; dCA3, 400 μm. (d and e) Optogenetic inhibition of the LC^TH^→dCA3 projection reduced CPP scores during the reinstatement test (d) and decreased locomotor velocity in response to DVC stimulation (e) in cocaine-CPP-trained mice. (f) Experimental timeline schematic for phasic/tonic activation of the LC^TH^→dCA3 circuit using optogenetics following VR-ABS treatment. (g) TH-Cre mice received AAV injections in the LC and were implanted with optical fibers above the dCA3. (h) Representative immunofluorescence images demonstrating ChR2-mCherry expression in LC^TH^ neurons and their terminals in dCA3. Scale bars: 200 μm. (i and j) Phasic, but not tonic, optogenetic activation of the LC^TH^→dCA3 circuit reversed the VR-ABS–induced reduction in CPP scores during the reinstatement test (i), and also increased locomotor velocity during DVC stimulation (j) in cocaine-CPP-trained mice. dCA1, dorsal Cornu Ammonis 1; dCA3, dorsal Cornu Ammonis 3; dDG, dorsal Dentate Gyrus; 4V, fourth ventricle. All data are presented as mean ± s.e.m.; statistical significance is indicated as ***p < 0.001.

We explored the possible outputs of LC^TH^ neurons involved in facilitating environmental cue-induced cocaine reinstatement. Utilizing a Cre-dependent anterograde AAV injection into the LC of TH-Cre mice, we observed EGFP-positive fibers in the dorsal hippocampus (dHip) (Fig. 5a–c). We aimed to discern which subregion within the dHip was influenced by LC^TH^ neurons in mediating cocaine reinstatement triggered environmental cues. Therefore, optical fibers were implanted into CA1 (dCA1), CA3 (dCA3), and DG (dDG) of the dHip of mice expressing NpHR in LC^TH^ neurons. Our results demonstrate that optogenetic inhibition of the LC^TH^→dCA3 circuit prevents cocaine reinstatement triggered by environmental cues in cocaine-CPP-trained mice (Fig. 5d). Furthermore, inhibition of this circuit also reduced the locomotor velocity in response to DVC stimulation in the VR environment (Fig. 5e). However, optogenetic inhibition of the LC^TH^→dCA1 or LC^TH^→dDG projections did not affect CPP scores or locomotion velocity during the reinstatement phase (Fig. S7a–f). These results indicate that the LC^TH^→dCA3 circuit is specifically required for environmental cue-induced cocaine reinstatement.

To evaluate whether the LC^TH^→dCA3 projection mediates the inhibitory effects of VR-ABS on cocaine reinstatement induced by environmental cues, we used a Cre-dependent viral vector expressing ChR2, which was injected into the LC of TH-Cre mice, along with optical fiber implantation in the dCA3 region (Fig. 5f–h). Phasic activation of the LC^TH^→dCA3 circuit, consisting of a series of ten 12-Hz pulses delivered at 0.5 Hz intervals, during the reinstatement phase reversed the suppression of cocaine reinstatement induced by VR-ABS treatment (Fig. 5i). Additionally, phasic activation of the LC^TH^→dCA3 projection increased locomotor velocity in response to DVCs in the VR environment in VR-ABS–treated mice (Fig. 5j). However, tonic activation (continuous stimulation at 3 Hz) did not produce these effects. These findings highlight the critical role of phasic activation of the LC^TH^→dCA3 circuit in cocaine reinstatement and suggest that VR-ABS prevents reinstatement by inhibiting this phasic activation.

### The augmentation of dynamic dopamine release from LC^TH^ neurons to dCA3 mediates cocaine reinstatement

**Figure 6.**
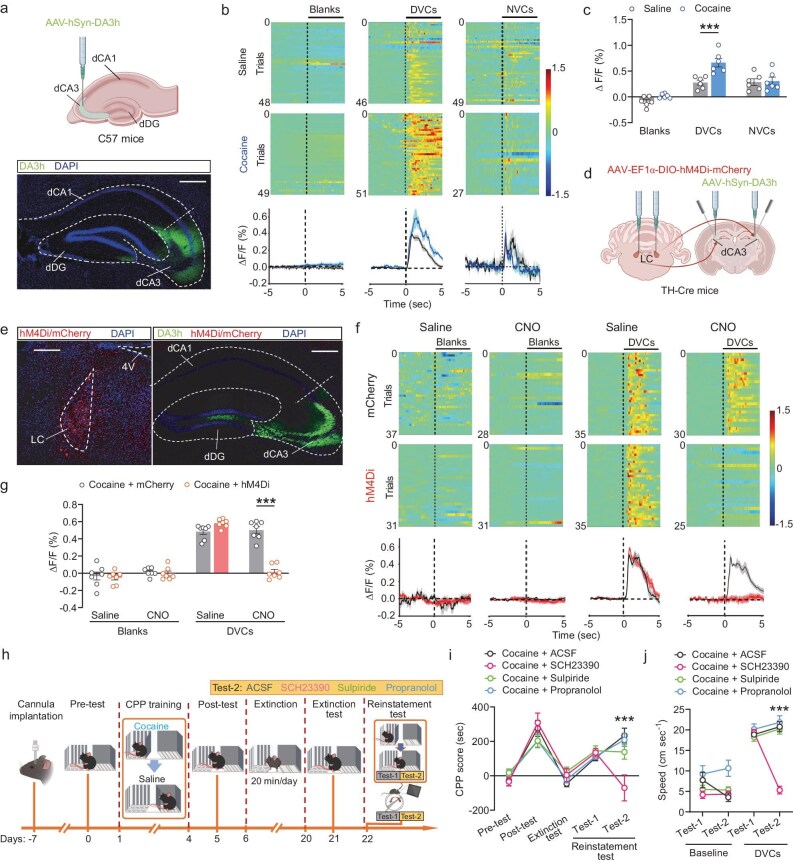
Enhanced dopamine release from LC^TH^ neurons to dCA3 promotes cocaine reinstatement. (a) Top: Mice received AAV injections in the dCA3 region and underwent optical fiber implantation above dCA3. Bottom: Representative images show DA3h expression in the dCA3 region. Scale bar: 400 μm. (b) Heatmaps and average traces illustrate the increased dopamine dynamics during exposure to DVCs in the VR environment within the dCA3 region. (c) Peak dopamine dynamics in dCA3 induced by DVCs in the VR environment were enhanced. (d) TH-Cre mice were injected with AAV in the LC and dCA3 regions and implanted with optical fibers above the dCA3. (e) Representative images show the expression of hM4Di-mCherry (red) in LC^TH^ neurons and DA3h (green) in dCA3 neurons. Scale bars: LC, 200 μm; dCA3, 400 μm. (f) Heatmaps and average transients show that chemogenetic inhibition of LC^TH^ neurons normalized the heightened dopamine dynamics in the dCA3 region induced by DVC stimulation during the reinstatement test in cocaine-CPP-trained mice. (g) Chemogenetic inhibition of LC^TH^ neurons also reversed the increased peak dopamine dynamics in the dCA3 induced by DVC stimulation during the reinstatement test in cocaine-CPP-trained mice. (h) Schematic illustration of the experimental protocol for microinjection of SCH23390 (4 μg/0.5 μl), sulpiride (4 μg/0.5 μl) or propranolol (4 μg/1 μl). (i and j) Microinjection of SCH23390 into the dCA3 reduced CPP scores during the reinstatement test (i) and decreased locomotor velocity in response to DVC stimulation (j) in cocaine-CPP-trained mice. The color scales on the right indicate Δ*F/F* values, and shaded regions around the means represent error bars. dCA1, dorsal Cornu Ammonis 1; dCA3, dorsal Cornu Ammonis 3; dDG, dorsal Dentate Gyrus. All data are presented as mean ± s.e.m.; statistical significance is indicated as ***p < 0.001.

Previous studies have highlighted the involvement of LC^TH^ neurons in spatial learning and memory by releasing dopamine and norepinephrine in the dHip [29–31]. However, the specific roles of dopamine and norepinephrine within the dHip in mediating cocaine reinstatement triggered by environmental cues remain unclear. To probe the real-time functions of dopamine and norepinephrine in this process, we utilized viral vectors to express genetically encoded fluorescent dopamine sensor (DA3h) and norepinephrine sensor (NE2h) in dCA3, dCA1, and dDG neurons, coupled with fiber photometry to monitor the *in vivo* dynamics of dopamine and norepinephrine release in these regions in the VR environment. We observed a significant increase in dopamine sensor signals specifically in the dCA3 region of cocaine-CPP-trained mice when exposed to DVCs in the VR environment during the reinstatement phase (Fig. 6a–c), while norepinephrine sensor signals remained unchanged (Fig. S8a–c). Meanwhile, there were no significant changes observed in the norepinephrine or dopamine sensor signals in the dCA1 or dDG regions (Fig. S8d–i; Fig. S9a–f). These results indicate a selective increase in dopamine sensor signals in the dCA3 of cocaine-CPP-trained mice specifically in response to DVC exposure during the reinstatement phase.

Moreover, to further validate the dopamine release originating from LC^TH^ neurons in dCA3, we utilized viral vectors to express hM4Di in LC^TH^ neurons and DA3h in dCA3 neurons of TH-Cre mice (Fig. 6d, e). Chemogenetic inhibition of LC^TH^ neurons in cocaine-CPP-trained mice prevented the DVC-induced elevation of dopamine signals in the dCA3 during the reinstatement phase, suggesting that DVCs enhance dopamine release in the dCA3 by activating LC^TH^ neurons (Fig. 6f, g). These findings suggest that exposure to DVCs in the VR environment triggers increased dopamine release from LC^TH^ projection neurons to the dCA3 during the reinstatement phase in cocaine-CPP-trained mice.

Subsequently, we applied pharmacological approaches to investigate the role of dopamine in the dCA3 in mediating cocaine reinstatement triggered by environmental cues (Fig. 6h). We found that microinjection of the D1 dopamine receptor antagonist SCH23390 into the dCA3 blocked environmental cue-induced cocaine reinstatement in cocaine-CPP-trained mice (Fig. 6j). Additionally, microinjection of SCH23390 reduced the locomotor velocity induced by DVCs in the VR environment in these mice (Fig. 6i). These results suggest that activation of the D1 receptor in dCA3 mediates the cocaine reinstatement induced by environmental cues.

### VR-ABS treatment suppresses the activation of SCi^CaMKIIα^→LC^TH^→dCA3 circuits

To enhance the visualization of connectivity within the SCi^CaMKIIα^→LC^TH^→dCA3 circuit, we employed a triple retrograde tracing strategy [32]. Specifically, retroAAV-hSyn-T2A-EGFP-Cre was injected into the dCA3 region, while Cre-dependent helper viruses were administered into the LC of C57 mice. Following 3 weeks, RV-EnvA-ΔG-DsRed was subsequently injected into the same site of LC, resulting in the observation of DsRed + neurons in the SCi region, thereby identifying a long-range SCi^CaMKIIα^→LC^TH^→dCA3 circuit (Fig. S10a, b).

To determine whether the SCi^CaMKIIα^→LC^TH^→dCA3 circuit mediates the inhibitory effect of VR-ABS on cocaine reinstatement triggered by environmental cues, we conducted *in vivo* fiber photometry to assess the impact of VR-ABS treatment on the activation of this neural pathway (Fig. S11a). The results showed that VR-ABS treatment reversed the elevated fluorescence intensity of GCaMP6s-expressing LC^TH^ neurons in response to DVCs during the reinstatement phase in cocaine-CPP-trained mice (Fig. S11b, c). Additionally, VR-ABS treatment reversed the heightened dopamine signal in the dCA3 region induced by DVCs in the VR environment during reinstatement (Fig. S11d, e). These combined findings indicate that VR-ABS treatment facilitates extinction by inhibiting the hyperactivation of the SCi^CaMKIIα^→LC^TH^→dCA3 neural circuitry in cocaine-CPP-trained mice.

## DISCUSSION

In this study, we introduced VR technology into rodent models of substance use disorder for the first time. This approach allowed us to precisely manipulate visual cues in the environment, where we observed that specific environmental visual cues robustly triggered cocaine reinstatement in cocaine-CPP-trained mice. Moreover, we uncovered a novel SCi^CaMKIIα^→LC^TH^→dCA3 neural circuit that mediates this cue-induced cocaine reinstatement. Leveraging the immersive nature of VR, we explored its potential for managing substance use disorder. Our findings demonstrated that combining VR with alternating bilateral sensory stimulation (VR-ABS) produced strong, long-lasting effects in preventing cocaine reinstatement. Specifically, VR-ABS treatment effectively inhibited cocaine reinstatement triggered by environmental cues by reducing hyperactivation of SCi^CaMKIIα^ neurons, thereby suppressing the SCi^CaMKIIα^→LC^TH^→dCA3 circuit (Fig. S12).

The use of VR technology in the medical field is expanding, with preliminary applications in diagnosing and treating mental disorders [33]. However, current VR treatment is limited by a lack of detailed research into specific intervention mechanisms [34]. In this study, we applied VR technology for the first time to explore the mechanisms underlying cocaine use disorder and to establish a research paradigm. By simulating the CPP environment through VR and precisely controlling experimental conditions, we gained deeper insights into behavior and neural responses, enhancing experiment repeatability and reliability. These findings lay essential groundwork for potential clinical applications. Nevertheless, discrepancies between VR activities and real-world behaviors persist [35]. Notably, we observed that animal movement speeds in the VR environment were higher than those in the real world, consistent with prior studies focusing on one-dimensional linear trajectories in VR [36]. This discrepancy may be attributed to animals’ enhanced freedom of movement in VR settings, unrestricted by physical space as in real-world environments. Improvements in scale accuracy and immersion of VR equipment are anticipated to address this issue and enhance experimental fidelity.

Although VR-based exposure strategies have shown some success in managing substance use disorders, such as reducing cravings for nicotine [37] and alcohol [10], their effectiveness in addressing methamphetamine use disorder has been limited [38]. This limitation is primarily attributed to the absence of standardized and personalized approaches in VR-based exposure interventions [39]. In response to this issue, we sought to integrate additional approaches into VR-based exposure interventions and found that VR-ABS effectively attenuates cocaine reinstatement. The eye movements induced by ABS effectively enhance memory retrieval and strengthen the efficacy of exposure treatment [40]. ABS has been applied in clinical treatments for conditions such as post-traumatic stress disorder, demonstrating significant efficacy in reducing conditioned fear memories [41]. While traditional ABS treatments have been explored in managing cocaine use disorder, the outcomes have been less than ideal [42]. Our research indicates that conventional ABS devices are less effective at reducing cocaine reinstatement compared to VR-ABS, primarily due to their inability to provide environments that facilitate extinction. Traditional ABS treatment relies on animals actively following light stimuli, which results in lower efficiency in inducing eye movements. In contrast, VR-ABS overcomes these limitations by utilizing the immersive nature of virtual reality to create environments that facilitate extinction. The head-fixed setup of the VR-ABS system enhances the efficiency of inducing eye movements and desensitization, thereby improving the effectiveness of extinction.

The SC brain region serves as a crucial treatment site for ABS treatment [13]. The SC is structurally organized into distinct functional and anatomical levels [43], with the intermediate (SCi) and deep (SCd) layers implicated in multisensory and motor functions, while the superficial layers (SCs) are primarily involved in visual processing [44]. Previous studies have demonstrated significant activation of SCs neurons during ABS stimulation [13], with inhibitory projections from SCs to SCi [44] potentially contributing to the reduction of SCi^CaMKIIα^ overactivation observed in VR-ABS treatment. However, further investigations are required to fully elucidate this mechanism. Given that SC neuron activity fluctuates during mouse wakefulness [45], all experiments in this study were conducted during the light cycle when normal mice exhibit reduced activity.

Prior studies have revealed that the SC provides synaptic input to LC^TH^ neurons [46]. Our further elucidation identified SCi^CaMKIIα^ neurons as projecting to LC^TH^ neurons. Interestingly, we observed that some SCi neurons that do not project to the LC were also activated in response to DVCs within the VR environment, suggesting that the SC may potentially mediate cocaine reinstatement through projections to other brain regions. The SC also projects to reward-related brain regions such as the ventral tegmental area (VTA) [47]. Previous research has implicated GABAergic projections from the SC to the VTA in regulating innate avoidance behaviors, social behavior orientation, and arousal induced by darkness [45,47,48]. However, whether the SC→VTA projection regulates cocaine reinstatement requires further investigation. Additional exploration is necessary to fully understand the role of the SC in mediating the circuitry underlying cocaine reinstatement.

LC^TH^ neurons co-release the neurotransmitters norepinephrine and dopamine [31,49]. However, we did not observe a specific increase in norepinephrine release in the dHip originating from LC^TH^ neurons upon exposure to DVCs. Despite ample pharmacological evidence suggesting that targeting α1-, α2-, and β-adrenergic receptors is a potential strategy to mitigate drug-seeking behavior and withdrawal symptoms [50], our findings indicate that applying the β-adrenergic antagonist propranolol in the dCA3 did not prevent the cocaine reinstatement triggered by environmental cues. This discrepancy may stem from differences in the site of norepinephrine action within the hippocampus and the timing of its involvement in different stages of cocaine use disorder. Utilizing fluorescence sensors to track the dynamic release of norepinephrine in distinct hippocampal subregions across various stages of cocaine use disorder could offer valuable insights into this phenomenon.

While VR technology has been applied in the treatment of substance use disorders, the lack of effective and standardized strategies and mechanistic research has become a bottleneck. Our study established a paradigm for investigating substance use disorders in rodents using VR technology and identified VR-ABS as an effective and long-lasting strategy for managing cocaine use disorder. Additionally, we uncovered the neural mechanism by which VR-ABS suppresses environmental cue-induced cocaine reinstatement, specifically through the inhibition of the SCi^CaMKIIα^→LC^TH^→dCA3 neural circuit. These findings provide valuable insights for developing novel approaches to managing cocaine use disorder.

## MATERIALS AND METHODS

The following animals were used in this study: C57BL/6 J (C57) mice were obtained from Hunan SJA Laboratory Animal Co. Ltd. (Changsha, China); TH-Cre mice were obtained from Jackson Laboratory (JAX:008601). Mice were bred and maintained in the Animal Resource Center of Tongji Medical College, Huazhong University of Science and Technology. Mice were housed in groups of four or five per cage and kept under standard laboratory conditions (12-hour light-dark cycle at a constant temperature of 22 ± 2°C with *ad libitum* food and water) unless otherwise indicated. Male and female mice, aged 8 to 10 weeks, were employed for the behavioral experiments. All animal procedures were conducted in accordance with the National Institutes of Health Guide for the Care and Use of Laboratory Animals and approved by the Animal Welfare Committee of Huazhong University of Science and Technology.

Detailed materials and methods are available in the Supplementary data.

## Supplementary Material

nwae467_Supplemental_Files

## Data Availability

All data needed to evaluate the conclusions in the paper are presented in the results and/or Supplementary materials. Any additional information is available from the corresponding author.

## RESOURCE AVAILABILITY

Further information and requests for resources and reagents should be directed to and will be fulfilled by the contact, Jian-Guo Chen (chenj@mails.tjmu.edu.cn) or Fang Wang (wangfanghust@hust.edu.cn).
