# Supplementary material for: A superior colliculus-originating circuit prevents cocaine reinstatement via VR-based eye movement desensitization treatment
Source: Natl Sci Rev. 2024 Dec 26;12(4):nwae467. doi: 10.1093/nsr/nwae467 (PMC11951104; doi:10.1093/nsr/nwae467)
Supplement: nwae467_Supplemental_Files [file nwae467_supplemental_files.zip › SI_clean_version_Liu_Y_NSR_1024_final_version.pdf]

Supplementary data

A Superior Colliculus-Originating Circuit Prevents Cocaine Reinstatement via VR-Based Eye Movement Desensitization Treatment

Yang Liu,[1,5] Zi-Xiang Zhou,[1,5] Qiu Lv, [1,5] Guan Huang,[1] Han Zhang, [1] Ye-Qin Wang,[1] Jian-Guo Chen, [1,2,3,4,*] and Fang Wang[1,2,3,4,6*]

[1]State Key Laboratory for Diagnosis and Treatment of Severe Zoonotic Infectious Diseases, Department of Pharmacology, School of Basic Medicine, Tongji Medical College, Huazhong University of Science and Technology, Wuhan, China.

[2]The Key Laboratory for Drug Target Researches and Pharmacodynamic Evaluation of Hubei Province, Wuhan, China.

[3]The Research Center for Depression, Tongji Medical College, Huazhong University of Science, 430030, Wuhan, China.

[4] Hubei Shizhen Laboratory, 430030, Wuhan, China.

[5]These authors contributed equally: Yang Liu, Zi-Xiang Zhou, Qiu Lv.

[6]Lead contact.

*Correspondence: wangfanghust@hust.edu.cn or chenj@mails.tjmu.edu.cn

This supplementary data file includes:

METHOD DETAILS

Supplementary Figures 1 to 12

Supplementary movie legends 1and 2

Supplementary Tables 1 to 4

METHOD DETAILS

Conditioned place preference (CPP)

For cocaine-conditioned place preference (CPP) training and testing, we utilized a three-compartment CPP apparatus (Yihong Technology, Wuhan, China) comprising two 15 × 15 × 20 cm^3 compartments with distinct tactile and visual cues, separated by a transparent compartment. The saline-paired compartment had black walls and a frosted perspex floor, while the cocaine-paired compartment had white walls with black vertical stripes and a wide-mesh patterned perspex floor. The compartments were connected by sliding doors within a soundproof box with top lighting. Animal exploration was recorded using the Any-Maze video-tracking system (Stoelting, Wood Dale, USA). Equal numbers of male and female mice were included in the study. After an initial pre-test phase, mice were randomly assigned to the cocaine CPP training group or saline control group. Cocaine CPP training spanned four consecutive days with morning and afternoon sessions. In the morning, mice were confined to the saline-paired compartment and received a saline injection (0.3 ml, i.p.). In the afternoon, mice were placed in the cocaine-paired compartment and received a cocaine injection (10 mg/kg, i.p.). Control mice received saline injections in both compartments. Following CPP training, a single post-test session was conducted to assess the preference level. Cocaine preference was evaluated using a CPP score calculated as the time spent in the cocaine-paired compartment minus time spent in the saline-paired compartment.

Extinction and environmental cue-induced reinstatement tests

After the acquisition of cocaine-induced conditioned place preference (CPP), mice underwent extinction training over a period of 14 consecutive days. During extinction sessions, mice were placed in the CPP apparatus for 20 minutes daily, with free access to both compartments of the chamber, but without receiving any cocaine or saline injections. Subsequently, on the following day, mice were placed in the neutral middle zone of the CPP apparatus and allowed to explore freely for 15 minutes to assess the extinction of cocaine memory.

Following successful extinction, defined as the absence of a significant difference in the time spent between the previously cocaine-paired and saline-paired compartments, mice underwent reinstatement tests. During these tests, mice were re-exposed to the environmental cues (both visual and tactile) that had been previously associated with cocaine, but no cocaine was administered. The reinstatement test lasted for 15 minutes, during which the time spent in the drug-paired compartment was recorded.

For the reinstatement test induced by singular contextual cues, cocaine-CPP-trained mice first underwent an extinction procedure to eliminate previously established place preferences. Following successful extinction, mice were re-exposed separately to either the singular tactile cue (wide-mesh floor) or the singular visual cue (white walls with black vertical stripes) that had been previously associated with cocaine, but no cocaine was administered. Each reinstatement test lasted for 15 minutes, during which the time spent in the drug-paired compartment was recorded.

For the reinstatement test with occluded visual input, cocaine-CPP-trained mice first underwent an extinction procedure to eliminate previously established place

preferences. Mice were habituated to wearing blindfolds for 20 minutes daily over three consecutive days prior to testing. After successful extinction, during re-exposure to the environmental cues previously associated with cocaine, without administering the drug, the experimental group was fitted with black plastic blindfolds to block visual input, while control mice wore transparent blindfolds to allow visual input. The test lasted for 15 minutes, during which the time spent in the cocaine-paired compartment was recorded to assess reinstatement behavior.

For the reinstatement test induced by different visual cues, cocaine-CPP-trained mice first underwent an extinction procedure to eliminate previously established place preferences. Following successful extinction, mice were separately re-exposed to drug-related visual cues (white walls with black vertical stripes) and non-drug-related visual cues (white walls with black horizontal stripes), without receiving cocaine. Each reinstatement test lasted for 15 minutes, during which the time spent in the drug-paired compartment was recorded to assess reinstatement behavior.

For the reinstatement test induced by localized visual cues, cocaine-CPP-trained mice first underwent an extinction procedure to eliminate previously established place preferences. Following successful extinction, mice were re-exposed separately to visual cues either confined to a 10 x 25 cm area or presented in full, both of which had been previously associated with cocaine, without administering the drug. Each reinstatement test lasted 15 minutes, during which the time spent in the cocaine-paired compartment was recorded to assess reinstatement behavior.

A significant increase in the time spent in this compartment compared to the extinction baseline was considered evidence of cue-induced cocaine reinstatement.

Virtual reality (VR)

The virtual reality (VR) system employed in this study was similar to the systems described previously. It consisted of a head-fixed setup with a projection screen, running wheel, and head-fixation apparatus. Mice were immobilized using a custom head-fixation device positioned above a running wheel, where locomotion was detected by a rotary encoder on the wheel axle connected to a microcontroller. Movement data were transmitted to a Raspberry Pi 4B computer running HallPassVR GUI software, facilitating real-time updates of the mouse's position within the VR environment and recording of movement speed. The VR environment was rendered on a Raspberry Pi 4 computer and projected onto a rear projection screen. We used the VR system to simulate a CPP apparatus environment at a 1:1 scale, allowing us to manipulate various visual cues within the system, including blanks, DVCs, and NVCs.

To record conditioned locomotor activity in response to visual cues during the reinstatement testing phase using the VR apparatus, cocaine-CPP-trained mice first underwent an extinction procedure to eliminate their previously established place preferences. Following extinction, the mice were given a 1-day adaptation period on the running wheel within the VR system. After adaptation, the mice proceeded to the VR reinstatement testing phase, which lasted for 15 minutes. During this phase, visual cue stimuli (blanks, DVCs, NVCs) were presented at the far end of the virtual CPP

environment as the mice navigated the virtual track. Their locomotor activity, measured by movement speed in response to the cues, was recorded. Upon reaching the end of the virtual track, the mice were teleported back to the start to continue testing.

ABS treatment

The ABS treatment apparatus for mice consisted of a perspex cylinder (7.5-cm radius, 40-cm height) featuring drug-related visual cues, including white walls with black vertical stripes. Additionally, the apparatus included a horizontal row of white LEDs (chip type, 12 W, 4-mm radius) positioned 5 cm above the floor. The cylinder was divided into four quadrants, each containing 8 LEDs spaced at 1-cm intervals. Mouse head direction was continuously monitored during treatments. LED lighting in a quadrant was manually activated when the mouse's head faced that direction, with only one quadrant active at a time. Within an active quadrant, LEDs sequentially flashed, creating a 1 Hz flashing pattern alternating from left to right. CPP-trained mice were confined in the ABS apparatus for 15 minutes, receiving visual stimulation once daily over 3 consecutive days. Mice were returned to their home cages for 14 days, followed by an extinction test.

VR extinction and VR-ABS treatments

For VR extinction, mice first underwent an extinction procedure to eliminate previously established place preferences. They began with a 1-hour adaptation period on the running wheel within the VR system. Afterward, mice were placed in the VR

apparatus for extinction training, during which visual cue stimuli (DVCs) were continuously presented. Upon reaching the end of the virtual environment, they were teleported back to the start. Each extinction session lasted 15 minutes daily for three consecutive days. Following the sessions, mice were returned to their home cages for 14 days, after which an extinction test was conducted.

For VR-ABS extinction, mice first underwent an extinction procedure to eliminate previously established place preferences, beginning with a 1-hour adaptation period on the running wheel within the VR system. During the VR-ABS phase, visual cues (DVCs) were displayed on the distant walls of the virtual CPP environment. As the mice approached the visual cue, the VR system transformed it into a moving white dot (1 cm in diameter) that moved either left or right at a speed of 1 cm/sec, prompting the mice to track its movement with their eyes. Once the white dot reached the edge of the virtual apparatus and disappeared, the mice were teleported back to the starting point. Training sessions lasted 15 minutes daily for three consecutive days. Afterward, mice were returned to their home cages for 14 days, followed by an extinction test.

Preparation of virus

The following viral constructs were obtained and packaged: AAV-CaMKIIα-GCaMP6s, AAV-CaMKIIα-hM4Di-mCherry, AAV-CaMKIIα-mCherry, AAV-EF1α-DIO-EGFP-T2A-TVA, AAV-EF1α-DIO-oRVG, AAV-EF1α-DIO-hM4Di-mCherry, AAV-EF1α-DIO-EGFP, AAV-CaMKIIα-ChR2-mCherry, AAV-CaMKIIα-NpHR-EGFP, AAV-EF1α-DIO-GCaMP6s, AAV-EF1α-DIO-GCaMP6s, AAV-EF1α-DIO-ChR2-mCherry,

AAV-EF1α-DIO-NpHR-EGFP, AAV-EF1α-DIO-hM4Di-mCherry, and RV-EnvA-ΔG-DsRed (RVdG; 2.00×10^10 colony forming units per ml). AAV-EF1α-DIO-mCherry-WPREs-pA was generated and packaged by Genechem (Shanghai, China). AAV-hSyn-DA3h and AAV-hSyn-NE2h plasmids were provided by Yulong Li (PKU-IDG/McGovern Institute for Brain Research, China) and packaged by BrainCase (Shenzhen, China). Retrograde tracing AAV viruses, retroAAV-hSyn-T2A-Cre and retroAAV-hSyn-T2A-EGFP-Cre, were generated and packaged by OBiO Technology (Shanghai, China). Detailed virus information is provided in Supplementary Table 1.

Stereotaxic surgery

For virus injection, mice were anesthetized with isoflurane (3.5% induction, 1.5%-2% maintenance) in oxygen and placed in a stereotaxic apparatus (RWD510, RWD Technology, Shenzhen, China). A scalp incision was made, and small volumes of AAVs (120-200 nl) were injected into specific brain areas at a rate of 40 nl/min using a micropump (World Precision Instruments, Sarasota, USA) with a controlled blunt needle. Injection coordinates were determined using the Paxinos and Franklin Mouse Brain Atlas, second edition, as follows: for SCi anteroposterior (AP), -4.00 mm; mediolateral (ML), ±1.00 mm; dorsoventral (DV), -1.95 mm; for LC: AP, -5.45 mm; ML, ±0.75 mm; DV, -3.60 mm; for dCA1: AP, -1.79 mm; ML, ±1.25 mm; DV, -1.50 mm; for dCA3: AP, -1.90 mm; ML, ±2.00 mm; DV, -2.10 mm; and for dDG: AP, -2.15 mm; ML, ±0.75 mm; DV, -2.10 mm. AAVs were delivered bilaterally, and the needle remained in place for ≥5 min to allow for virus diffusion. Following closure of the

incision with sutures, mice were allowed to recover in their home cages for approximately 3 weeks.

For rabies input tracing, a 100 nl mixture of AAV-EF1α-DIO-EGFP-T2A-TVA-WPREs-pA and AAV-EF1α-DIO-oRVG-WPREs-pA (1:1) was injected into LC of TH-Cre. After two weeks, RV-EnvA-ΔG-DsRed (200 nl) was injected into LC. Mice were housed in a P2 laboratory for 7 days.

For the implantation of optical fibers (200 μm diameter, 0.37 NA), drug cannulas (26GA stainless steel), and four platinum-iridium-coated HM-L tetrodes (17 mm in length with 20% platinum content; California Fine Wire Company, Grover Beach, USA), these devices were surgically implanted into specific brain regions, including the SCi, LC, dCA1, dCA3, and dDG in mice. The devices were carefully advanced into the target areas using a piezoelectric micromanipulator (Scientifica, East Sussex, UK). Once positioned, the micro-drive assembly was securely attached to the skull using jewelry screws and dental cement. Mice were housed for 5-7 days post-surgery before proceeding to the next experimental steps.

Fiber photometry

For in vivo photometry recording, we utilized the FiberOptoMeter system developed by ThinkerTech Nanjing Bioscience Inc, Nanjing, China. This setup employed a continuous blue LED (470 nm) as the excitation light source, with laser power adjusted to 20 μW at the fiber tip to minimize photobleaching. Fluorescence emitted by GCaMP6s was collected and converted into voltage signals, which were digitized at 50

Hz using the Fiber photometry software Tripple-Col-NANOout. Additionally, infrared camera recordings were synchronized with acute neural responses during visual cue stimulation and animal behavior. Following surgery, mice were individually housed and given a 3-day recovery period before beginning CPP training. Cocaine-CPP-trained mice then underwent an extinction procedure to eliminate previously established place preferences. In vivo photometry recordings were subsequently conducted during the VR visual cue-induced reinstatement test. During VR testing, mice were first acclimated to the VR environment for 10 minutes before baseline measurements were taken. Visual cue stimuli (DVCs and NVCs) were then presented sequentially at the far end of the virtual environment while conducting photometry recordings.

OpSignal software (ThinkerTech Nanjing Bioscience Inc, Nanjing, China) analyzed photometry data, segmented by behavioral events within trials. ΔF/F values were calculated as (F-F0) / F0, with F0 representing baseline fluorescence averaged over a 2-5 second control time window preceding trigger events. Heatmaps and average plots visualized ΔF/F values, with shaded areas representing standard error of the mean.

Patch-clamp recording

For brain tissue preparation, mice were anesthetized with isoflurane (3.5% induction, 1.5%-2% maintenance) and transcardially perfused with 20 ml of ice-cold, oxygenated cutting solution containing 1.3 mM ascorbate acid, 120 mM choline chloride, 2.6 mM KCl, 0.5 mM $CaCl_2$, 7 mM $MgCl_2$, 26 mM $NaHCO_3$, 1.25 mM NaH_2PO_4, and 15 mM glucose. The brain was quickly extracted and sectioned into 300 μm coronal slices

containing the SC or LC using a semiautomatic vibrating blade microtome. Following this, the slices were incubated at 28°C for 30 min in a modified artificial cerebrospinal fluid (ACSF) containing: 2.5 mM KCl, 1.25 mM NaH_2PO_4, 26 mM $NaHCO_3$, 10 mM glucose, 6 mM Mg_2SO_4, 210 mM sucrose, and 0.5 mM $CaCl_2$ under carbogenation (pH 7.35 when saturated with 95% O_2/ 5% CO_2). Whole-cell patch-clamp recording was performed using an upright fixed-stage microscope equipped with a water immersion objective (40×, 0.8 numerical aperture). Pipettes with a resistance of 3 to 5 megohms were used for recording.

For action potential (AP) firing recordings, EGFP-labeled $SCi^{CaMKII\alpha}$ neurons in mice were selected. Current-clamp recordings were conducted using an internal solution composed of 97 mM K-gluconate, 38 mM KCl, 4 mM ATP-Mg, 7 mM creatine phosphate disodium salt tetrahydrate, 0.35 mM GTP-Na_2, 0.35 mM EGTA, 6 mM NaCl, and 20 mM HEPES (pH 7.25). Current injections were applied with 50-pA increments ranging from 0 to 350 pA to assess intrinsic neuronal excitability.

For mEPSC recording, EGFP+ $SCi^{CaMKII\alpha}$ neurons in mice were targeted. Patch pipettes were filled with an internal solution containing 4 mM ATP-Na_2, 0.5 mM GTP-Na_2, 10 mM creatine phosphate disodium salt tetrahydrate, 7.5 mM CsCl, 127.5 mM cesium methanesulfonate, 0.6 mM EGTA, 2.5 mM $MgCl_2$, and 10 mM HEPES at pH 7.35. During mEPSC recordings, cells were voltage-clamped at -70 mV in the presence of 30 μM bicuculline and 1 μM tetrodotoxin (TTX). Miniature events were recorded 5 minutes after establishing the whole-cell patch-clamp recording mode.

For recording oEPSCs, patch pipettes were filled with the same internal solution used for recording mEPSCs. TH-Cre mice expressing EGFP in LC^{TH} neurons and ChR2 in $SCi^{CaMKII\alpha}$ neurons were utilized. LC^{TH} neurons were clamped at -70 mV and received brief blue light pulses (5 ms) at 15-second intervals. Monosynaptic currents were recorded in the presence of sodium and potassium channel blockers (1 μM TTX and 100 μM 4-AP, respectively). To induce phasic firing of LC^{TH} neurons. Blue light (473 nm wavelength, 3 mW power, 10-ms pulse width for 500 ms duration) was delivered at 10 Hz to stimulate $SCi^{CaMKII\alpha}$ terminals. Concurrently, we continuously recorded action potentials in LC^{TH} neurons. Phasic firing detection and determination were performed using NeuroExplorer 5 software.

All recordings were conducted with a MultiClamp700B amplifier (Molecular Devices, Sunnyvale, USA). Analog signals were low-pass–filtered at 2 kHz, digitized at 20 kHz using Digidata 1440A (Molecular Devices, Sunnyvale, USA), and recorded using pClamp 10 software (Molecular Devices, Sunnyvale, USA). Detailed chemical information used in the experiment is provided in Supplementary Table 2.

In vitro electrophysiology

For in vivo multi-tetrode recording, electrodes were surgically implanted into the SCi or LC of mice. Following surgery, mice were individually housed and given a 3-day recovery period before starting CPP training. Cocaine-CPP-trained mice then underwent an extinction procedure to eliminate previously established place preferences. Afterward, they underwent a 2-day adaptation period with a tethered patch

cord in the VR testing apparatus. Subsequently, in vivo multi-tetrode recordings were conducted during the VR visual cue-induced reinstatement test. During recording sessions, electrodes were connected to the recording equipment through AC-coupled unity-gain operational amplifiers (Plexon, Dallas, USA), providing signal amplification ranging from 4000 to 8000-fold. Spikes were recorded and isolated using a 250-Hz low-pass filter and a 250-Hz high-pass filter with commercial software, OmniPlex (Plexon, Dallas, USA).

Multi-tetrode recording signals were analyzed using Offline Sorter (Plexon, Dallas, USA) for spike sorting. Units were assessed based on isolation distance and L-ratio, with exclusion criteria of isolation distance < 20 or L-ratio > 0.1. NeuroExplorer 5 software (Nex Technologies, Colorado Springs, USA) detected phasic firing in LC^{TH} neurons using specific parameters. LC^{TH} neurons were identified by characteristics including action potential width ≥ 1.1 ms, slow firing rate (<12 Hz), and specific firing patterns. Basal firing rate and bursting rate were calculated within 120 seconds after a stable 5-minute baseline period. Bursting activity was analyzed using burst score (bursting event frequency × number of spikes per burst). Phasic bursts were defined as two spikes with an interspike interval of less than 80 ms.

Optogenetic/chemogenetic stimulation

For optogenetic stimulation during behavioral experiments, we used a 473-nm (blue light) or 589-nm (yellow light) laser system (AniLab Software & Instruments, Ningbo, China) connected to the optical fiber implanted in the mouse via a patch cord. To

activate the SCi$^{CaMKII\alpha}$→LC circuit, we delivered 473-nm blue light in 10-ms pulses at 10 Hz. Phasic activation of LCTH→dCA3 circuits involved ten 12 Hz pulses with 0.5 Hz intervals. Tonic activation was achieved by continuous 3 Hz stimulation. For optogenetic inhibition, yellow light was continuously delivered at 5 mW, with the laser output controlled by a function signal generator (Newdoon Technology, Hangzhou, China).

For chemogenetic inhibition of SCi$^{CaMKII\alpha}$ (or SCi$^{CaMKII\alpha}$→LC projection) or LCTH neurons, we administered CNO (0.5 mg/kg) to CaMKIIα-hM4Di or TH-hM4Di mice via i.p. injection 30-60 min before CPP or voluntary running wheel tests, respectively. Brain sections were collected to verify virus expression in all experiments.

Drugs and treatments

For chemical administration, mice with implanted drug cannulas were anesthetized using isoflurane (3.5% induction, 1.5-2% maintenance) in oxygen. We microinjected D1 dopamine receptor blocker SCH23390 (4 μg/0.5 μl) obtained from Tocris (Bristol, UK), D2 dopamine receptor blocker Sulpiride (4 μg/0.5 μl) from Sigma (St. Louis, USA), or norepinephrine receptor blocker Propranolol (4 μg/1 μl) from Sigma (St. Louis, USA) into the dCA3 region using a 10-μl syringe and a blunt needle, controlled by a micropump (World Precision Instruments, Sarasota, USA) at a rate of 100 nl/min. After 30 min, behavior tests were conducted. Detailed drug information is provided in Supplementary Table 2.

Histology and immunohistochemistry

For immunofluorescence analysis, mice were euthanized 60-90 minutes after a 15-minute visual cue stimulus following isoflurane anesthesia (3.5% induction, 1.5%-2% maintenance). Transcardial perfusion was performed using cold PBS followed by ice-cold 4% paraformaldehyde (PFA) in PBS for 3 minutes. Brains were then post-fixed in 4% PFA at 4°C overnight and equilibrated in 30% sucrose. Brain sections of 35-μm thickness were obtained using a cryostat microtome.

The sections were washed with PBS and then blocked with 0.3% Triton X-100 and 10% normal goat serum in PBS for 1 hour, followed by overnight incubation with primary antibodies at 4°C. Primary antibodies used included anti-c-Fos (rabbit, 1:1000, ab190289, Abcam), anti-CaMKIIα (mouse, 1:100, 50049, Cell Signaling Technology), anti-c-Fos (mouse, 1:200, sc-166940, Santa Cruz Biotechnology), and anti-VGLUT1 (rabbit, 1:100, 135 302, Synaptic Systems). After rinsing with PBS, the sections were incubated with fluorescence-conjugated secondary antibodies. Detailed information about the antibodies used in the experiment is provided in Supplementary Table 3.

Mounted sections were stained with DAPI and then imaged and analyzed using a laser scanning confocal microscope (FV1000, OLYMPUS, Tokyo, Japan). To assess SCi neuron activation, c-Fos-immunoreactive neurons in specific brain regions were quantified from 3 coronal slices per mouse (n = 3 mice/group). Automated cell counting was performed using Imaris V8.1.0 software. Data from the same experimental group were pooled for statistical analysis, which was performed in a blinded manner.

Quantification and statistical analysis

The minimum sample size was determined based on experimental requirements. Data are presented as means ± SEM and visualized using GraphPad Prism v.8.0.2 (GraphPad Software, San Diego, USA). Statistical analyses were performed using SPSS 18.0 software (IBM, Armonk, USA). Paired or unpaired two-tailed t-tests were employed for relevant comparisons, while one-way or two-way ANOVA followed by post-hoc Bonferroni's tests were used for multiple group analyses. Statistical significance was set at $p < 0.05$. Detailed statistical analyses for each figure are provided in Supplementary Table 4.

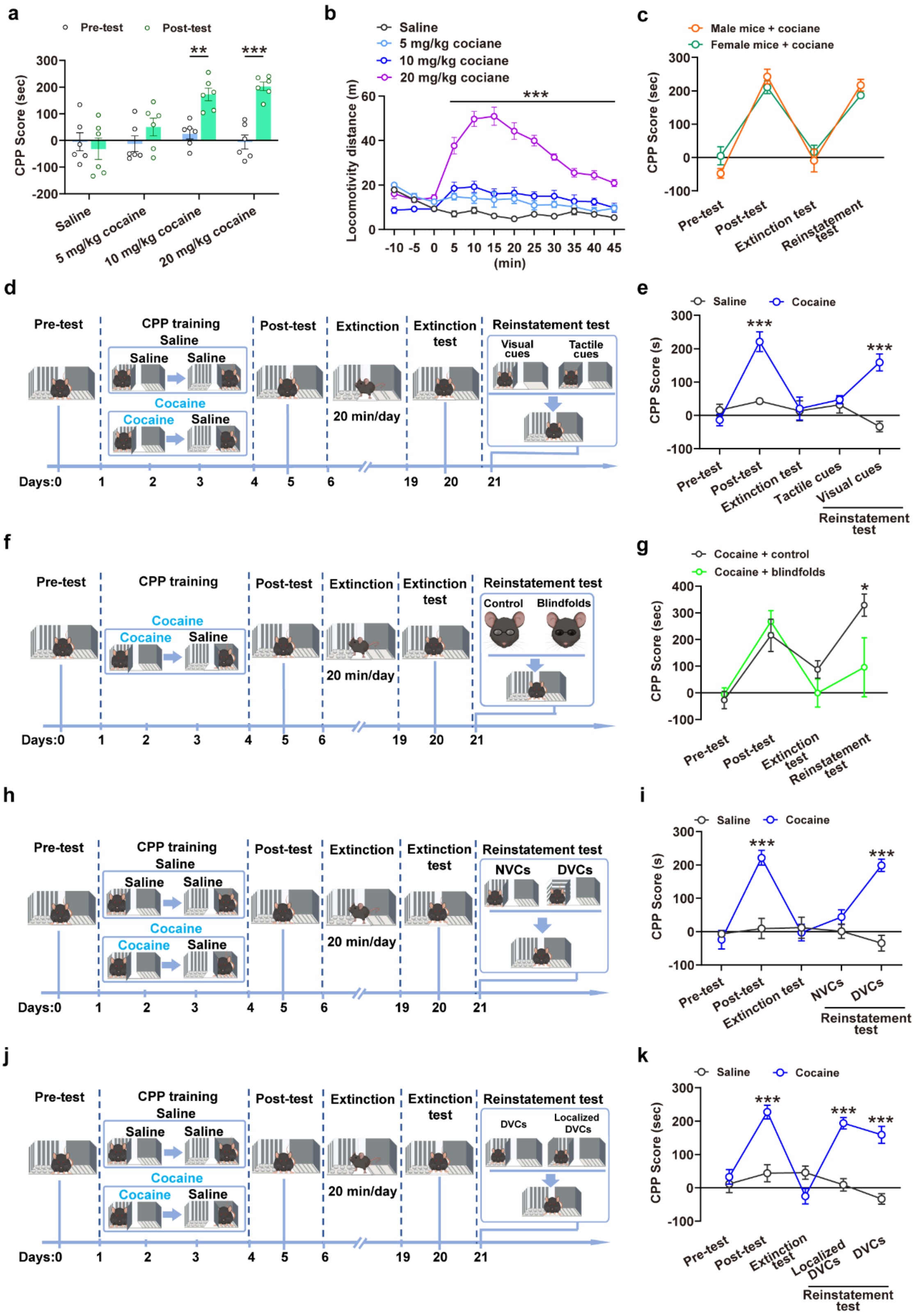

Supplementary Figure 1. Environmental visual cues play a critical role in triggering cocaine reinstatement.

(a) CPP training with both 10 mg/kg and 20 mg/kg of cocaine effectively induced place

preference. **(b)** 20 mg/kg cocaine increased locomotive distance. **(c)** No significant differences were observed between male and female mice during CPP training, extinction, and reinstatement phases. **(d)** Experimental timeline for inducing cocaine reinstatement tests conducted in chambers with either single visual or tactile cues. **(e)** Single visual cues significantly increased CPP scores during reinstatement tests in cocaine-CPP-trained mice. **(f)** Experimental design assessing the effects of blocking visual input with blindfolds on cocaine reinstatement. **(g)** Blocking visual input with blindfolds prevented the increase in place preference during reinstatement tests in cocaine-CPP-trained mice. **(h)** Experimental design demonstrating the effect of different visual cues on cocaine reinstatement. **(i)** Cocaine-CPP-trained mice exposed to DVCs showed increased CPP scores compared to those exposed to NVCs during reinstatement tests. **(j)** Experimental design illustrating the effect of localized DVCs on cocaine reinstatement. **(k)** Re-exposure to localized DVCs also increased CPP scores during reinstatement tests in cocaine-CPP-trained mice. All data are presented as mean ± s.e.m.; Statistical significance indicated as $^{**}p < 0.01$ and $^{***}p < 0.001$.

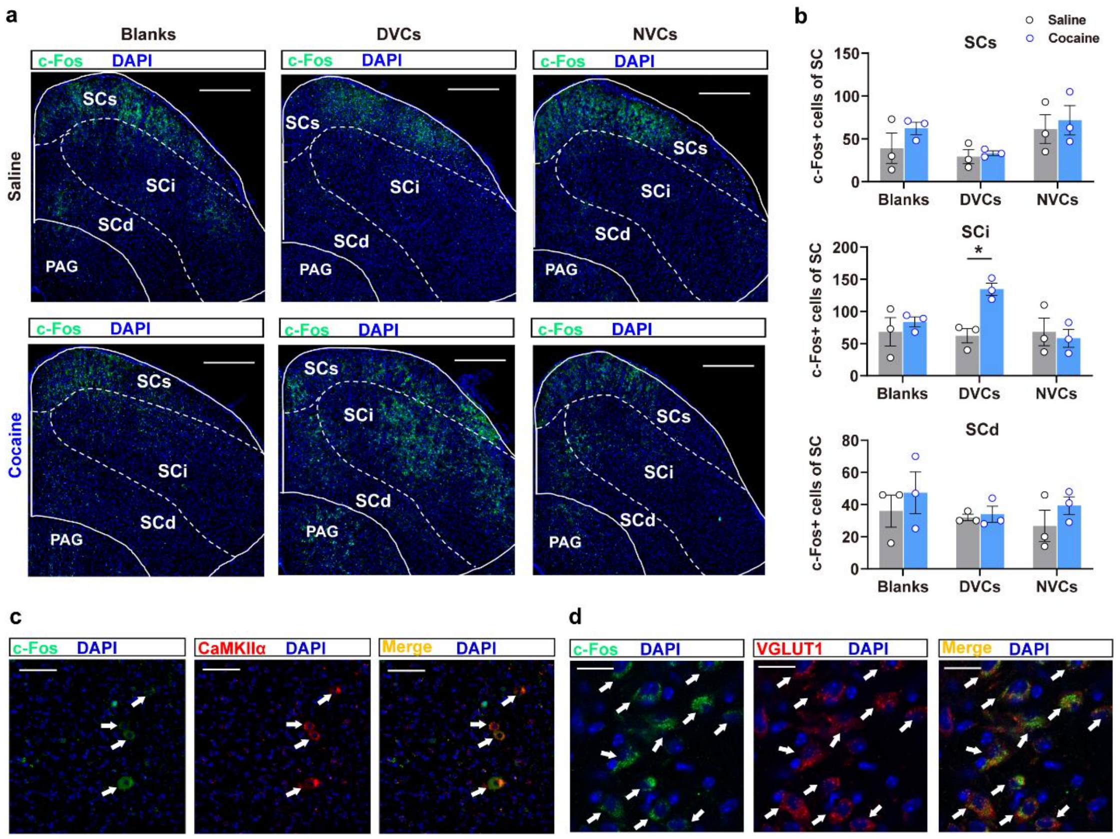

Supplementary Figure 2. Enhanced responsiveness of SCi$^{CaMKII\alpha}$ neurons to environmental cues.

(a) Representative images showing c-Fos immunofluorescence. Scale bars: 500 μm. **(b)** Increased c-Fos expression was observed in the SCi subregion, but not in the SCd or SCs, of cocaine-CPP-trained mice following DVC stimulation. **(c)** Representative immunofluorescence images demonstrating co-localization of c-Fos (green) with CaMKIIα (red)-positive neurons. Scale bar: 50 μm. **(d)** Representative immunofluorescence images showing co-localization of c-Fos (green) with VGLUT1 (red)-positive neurons. Scale bar: 25 μm. All data are presented as mean ± s.e.m.; Statistical significance indicated as $*p < 0.05$.

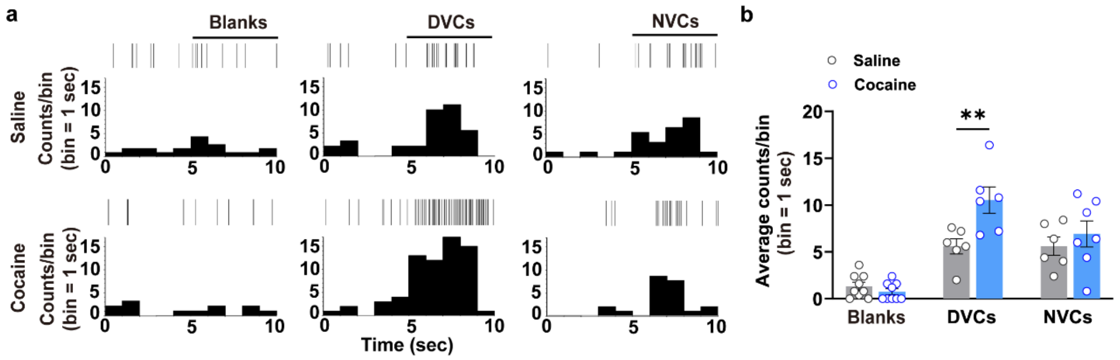

Supplementary Figure 3. Enhanced activation of SCi neurons in response to environmental cues in cocaine-CPP-trained mice.

(a) Raster plots and spike time histograms depict firing activity of SCi neurons in response to visual cues, presented as neuronal counts per second. **(b)** Enhanced firing activity in response to DVCs was observed in the SCi neurons of cocaine-CPP-trained mice. All data are presented as mean ± s.e.m.; Statistical significance indicated as $^{**}p < 0.01$.

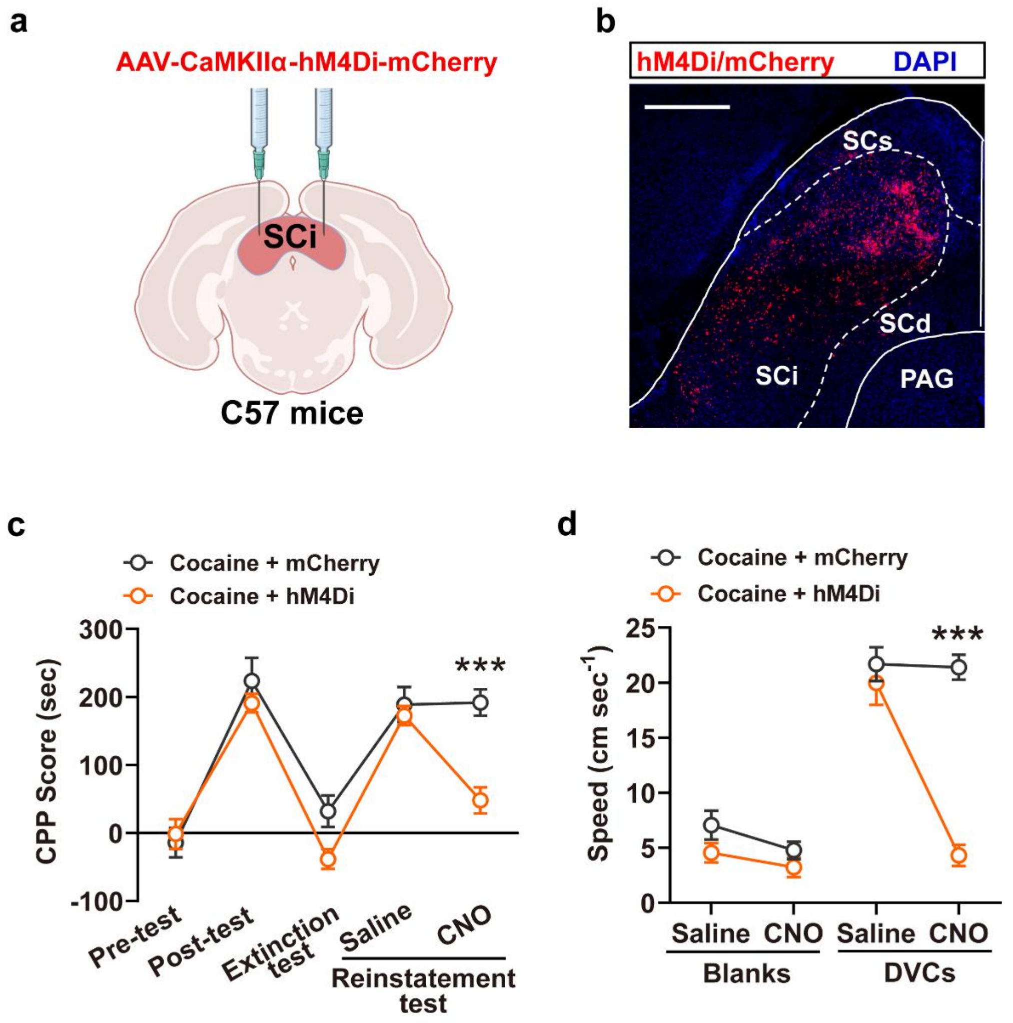

Supplementary Figure 4. Chemogenetic silencing of $SCi^{CaMKII\alpha}$ neurons prevents cocaine reinstatement triggered by environmental cues

(a) Schematic diagram illustrating AAV injection into SCi. **(b)** Representative immunofluorescence images showing hM4Di-mCherry expression. Scale bars: 500 μm. **(c, d)** Chemogenetic inhibition of $SCi^{CaMKII\alpha}$ neurons reduced CPP scores during the reinstatement test (c) and decreased locomotor velocity in response to DVC stimulation (d) in cocaine-CPP-trained mice. SCs, superficial layers of the superior colliculus; SCi, intermediate layers of the superior colliculus; SCd, deep layers of the superior colliculus. All presented data are expressed as mean ± s.e.m.; Statistical significance indicated as ***$p < 0.001$.

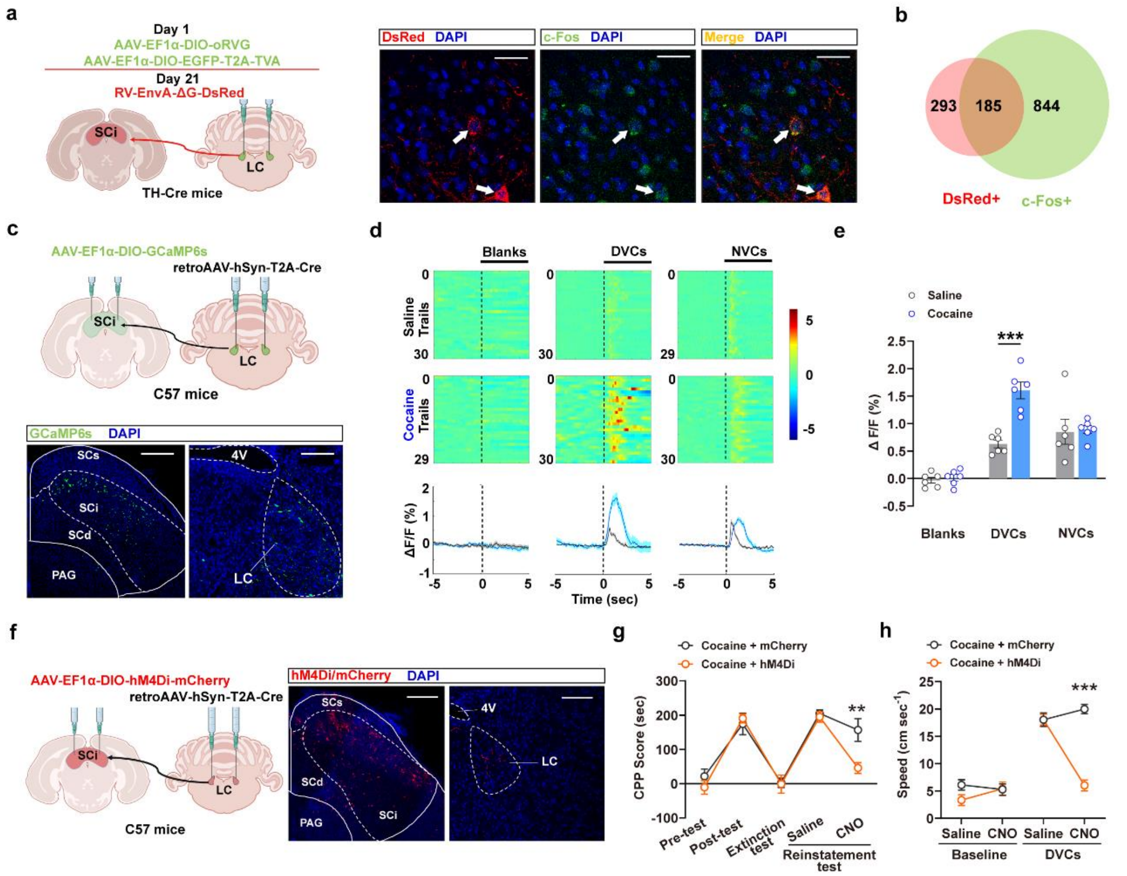

Supplementary Figure 5. Chemogenetic silencing of SCi→LC projection neurons suppressed environmental cue-induced cocaine reinstatement.

(a) Representative immunofluorescence images showing co-localization of c-Fos (green) with DsRed-positive neurons retrogradely labeled from the LC region to the SCi subregion. Scale bar: 25 µm. **(b)** In cocaine-CPP-trained mice, among 293 neurons retrogradely labeled from the LC to the SCi, 185 were activated by DVCs, accounting for 63.1% of the total. **(c)** Top: Schematic diagram illustrating retrograde AAV injection in the LC and AAV injection into the SCi. Bottom: Representative immunofluorescence images showing GCaMP6s expression in SCi neurons projecting to the LC. Scale bars: SCi, 500 µm; LC, 100 µm. **(d)** Heatmaps and average calcium transients showing enhanced responses to DVCs during the reinstatement test in cocaine-CPP-trained mice.

Shaded areas indicate error margins. The color scale on the right represents ΔF/F values. **(e)** Peak calcium transients in response to DVCs during the reinstatement test were significantly increased in cocaine-CPP-trained mice. **(f)** Left: Schematic diagram showing retrograde AAV injection into the LC and AAV injection into the SCi. Right: Representative immunofluorescence images illustrating hM4Di-mCherry expression in SCi neurons projecting to the LC. Scale bars: SCi, 500 μm; LC, 200 μm. **(g, h)** Chemogenetic inhibition of SCi→LC projection neurons reduced CPP scores during the reinstatement test (g) and decreased locomotor velocity in response to DVC stimulation (h) in cocaine-CPP-trained mice. SCs, superficial layers of the superior colliculus; SCi, intermediate layers of the superior colliculus; SCd, deep layers of the superior colliculus; PAG, periaqueductal gray; 4V, fourth ventricle. All presented data are expressed as mean ± s.e.m.; Statistical significance indicated as $**p < 0.01$ and $***p < 0.001$.

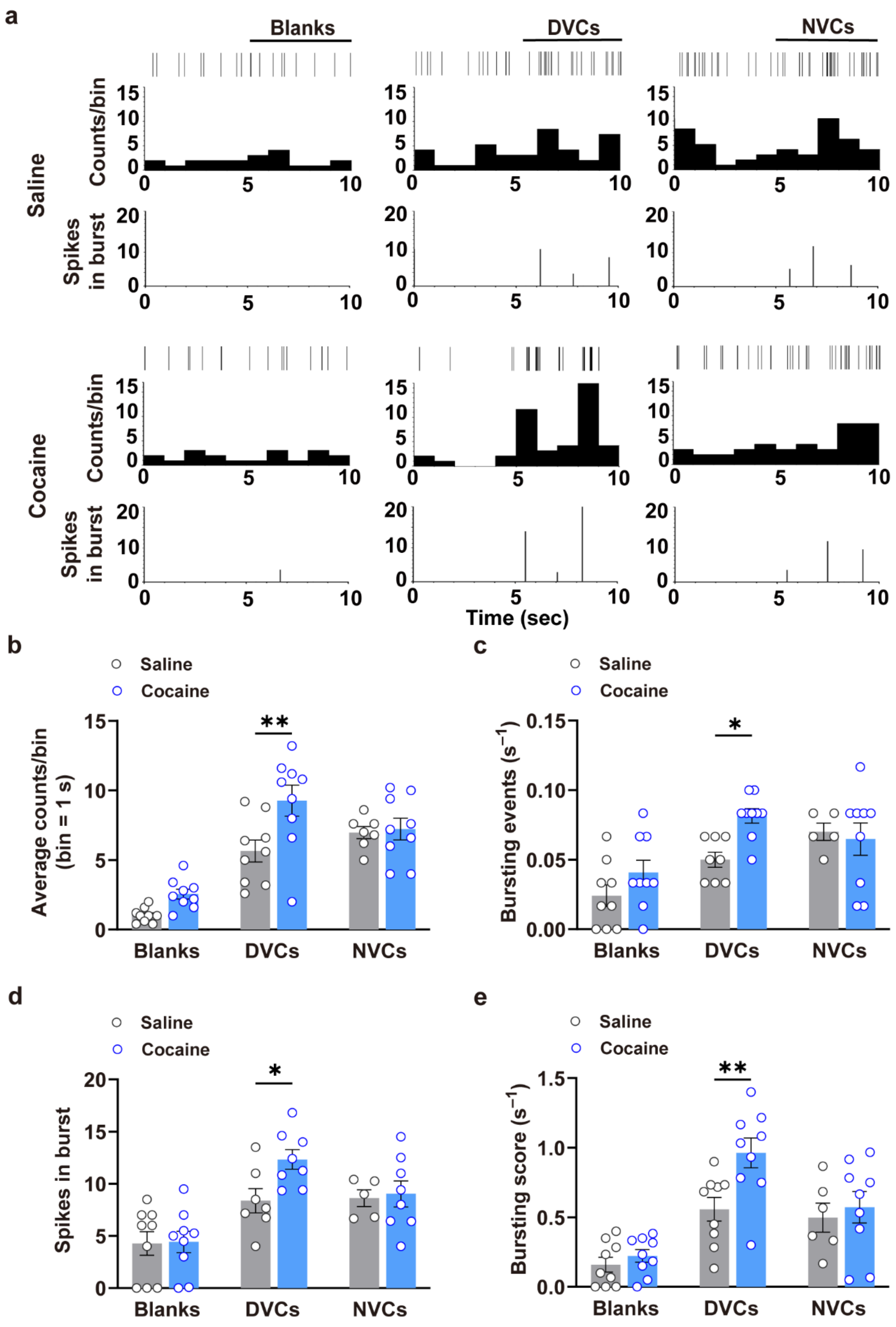

Supplementary Figure 6. Enhanced phasic activation of LC neurons in response to environmental cues.

(a) Raster plots and spike time histograms illustrate LC neuron firing responses and phasic burst spike counts. **(b)** Cocaine-CPP-trained mice displayed increased firing activity of LC neurons in response to DVCs. **(c)** Cocaine-CPP-trained mice showed enhanced bursting events of LC neurons in response to DVCs. **(d)** Cocaine-CPP-trained mice exhibited more spikes per burst of LC neurons in response to DVCs. **(e)** Cocaine-CPP-trained mice demonstrated a higher bursting score of LC neurons in response to DVCs. All data are presented as mean ± s.e.m.; Statistical significance indicated as $*p < 0.05$ and $**p < 0.01$.

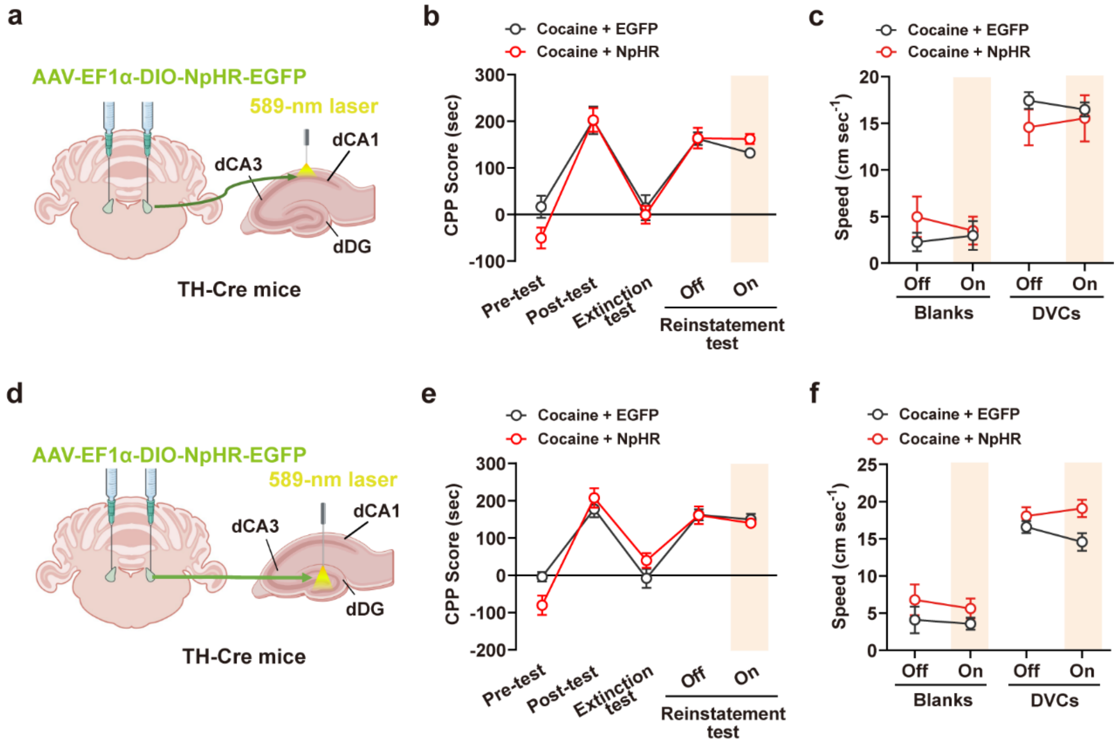

Supplementary Figure 7. Optogenetic inhibition of the LC^{TH}→dCA1 or LC^{TH}→ dDG projections failed to induce cocaine reinstatement..

(a) Mice received AAV injections in the LC followed by optical fiber implantation above the dCA1 region. **(b, c)** Chemogenetic inhibition of the LC^{TH}→dCA1 projection did not reduce CPP scores during the reinstatement test (b), nor did it decrease locomotor velocity in response to DVC stimulation (c) in cocaine-CPP-trained mice. **(d)** Following AAV injection into the LC, mice received optical fiber implantation above the dDG region. **(e, f)** Chemogenetic inhibition of the LC^{TH}→dDG projection did not reduce CPP scores during the reinstatement test (e), nor did it decrease locomotor velocity in response to DVC stimulation (f) in cocaine-CPP-trained mice. All presented data are expressed as mean ± s.e.m.

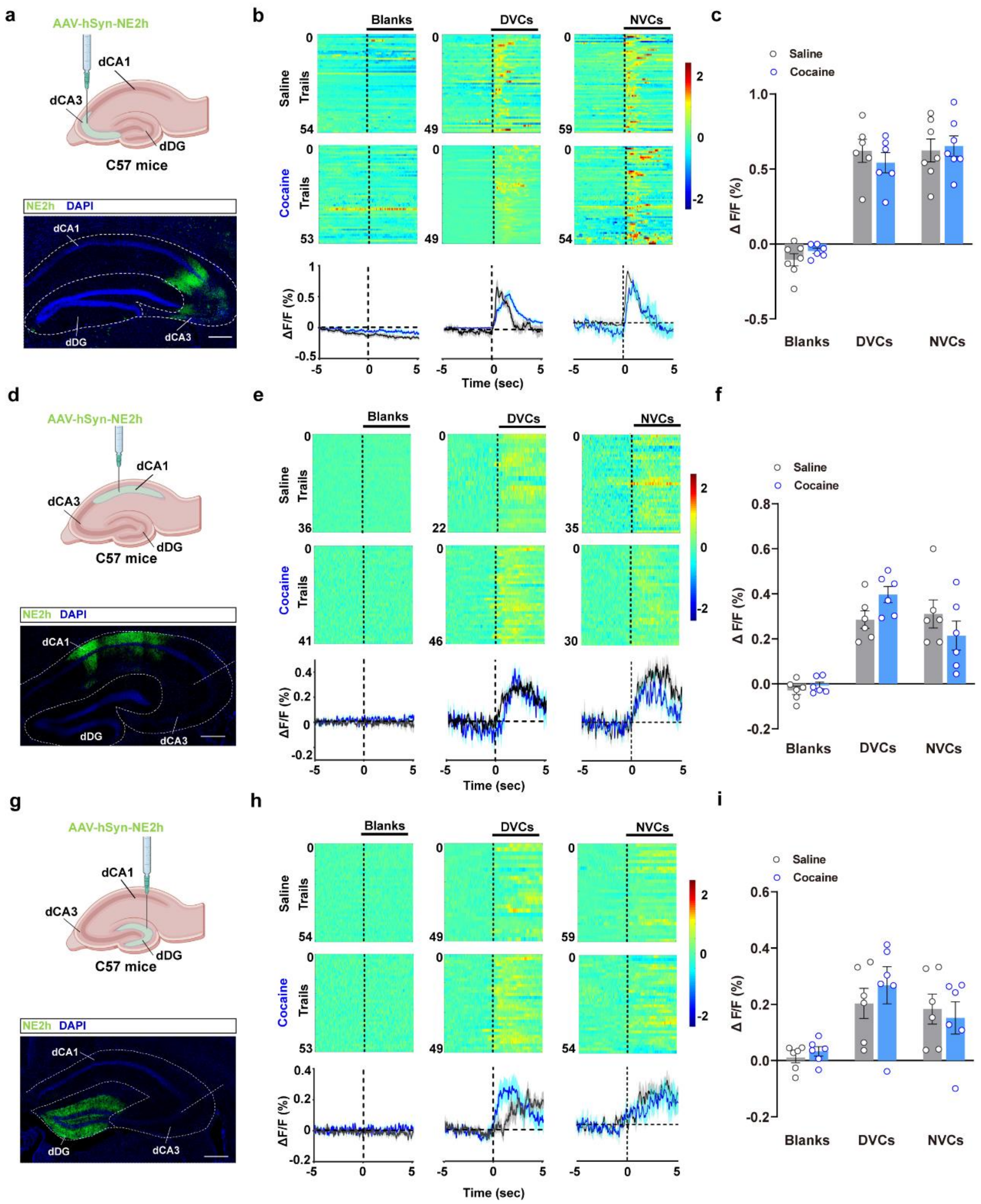

Supplementary Figure 8. Absence of changes in norepinephrine dynamics in the dCA3, dCA1, or dDG regions in response to environmental cues during the reinstatement phase in cocaine-CPP-trained mice.

(a) Schematic and representative images showing NE2h expression in dCA3. Scale bar, 400 μm. **(b, c)** Heatmaps and average traces demonstrate that norepinephrine dynamics in the dCA3 remained unchanged in response to DVCs during the reinstatement phase

in cocaine-CPP-trained mice (b). Peak norepinephrine dynamics in the dCA3 showed no significant changes in response to DVCs during this phase (c). **(d)** Schematic and representative images displaying NE2h expression in dCA1. Scale bar, 400 μm. **(e, f)** Heatmaps and average traces demonstrate that norepinephrine dynamics in the dCA1 remained unchanged in response to DVCs during the reinstatement phase in cocaine-CPP-trained mice (e). Peak norepinephrine dynamics in the dCA1 showed no significant changes in response to DVCs during this phase (f). **(g)** Schematic and representative images demonstrating NE2h expression in dDG. Scale bar, 400 μm. **(h, i)** Heatmaps and average traces demonstrate that norepinephrine dynamics in the dDG remained unchanged in response to DVCs during the reinstatement phase in cocaine-CPP-trained mice (h). Peak norepinephrine dynamics in the dDG showed no significant changes in response to DVCs during this phase (i). Shaded areas around the means represent error bars. Color scales on the right indicate ΔF/F. dCA1, dorsal Cornu Ammonis 1; dCA3, dorsal Cornu Ammonis 3; dDG, dorsal Dentate Gyrus. All presented data are expressed as the mean ± s.e.m.

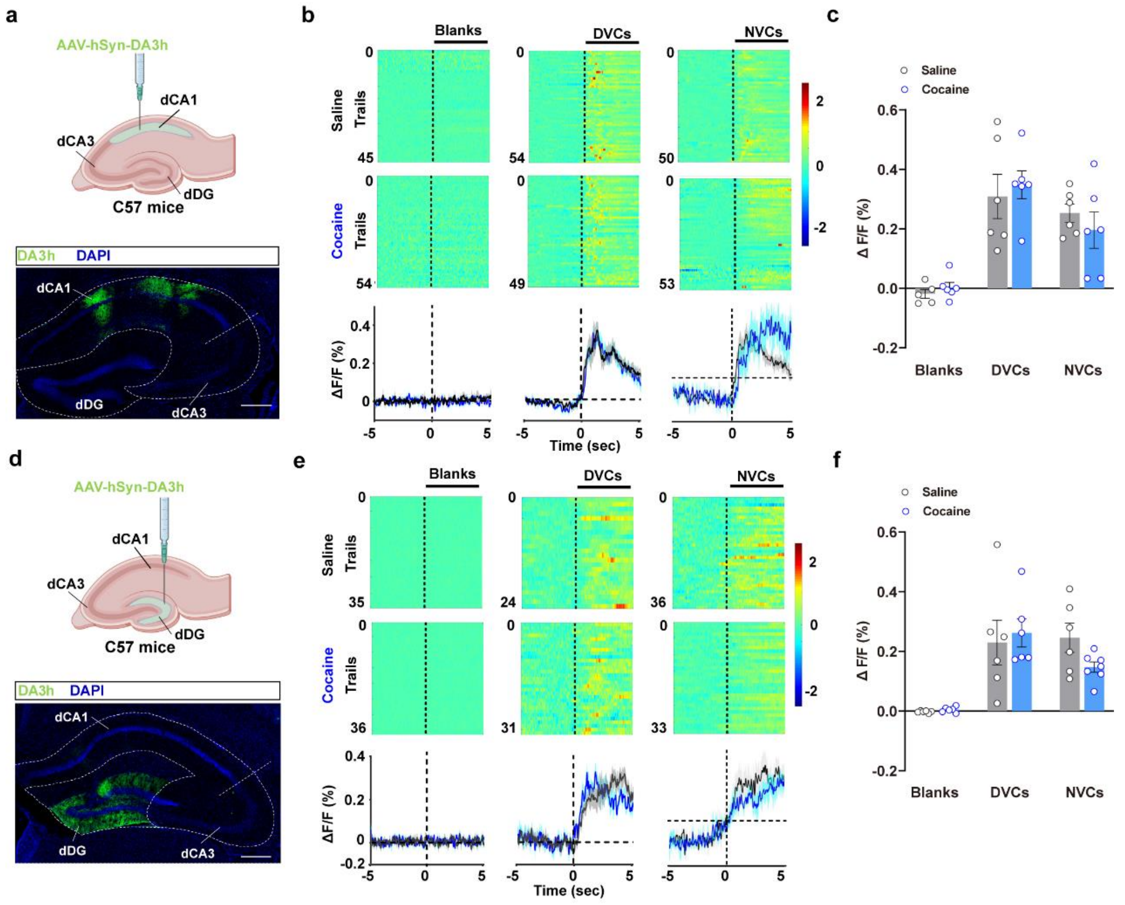

Supplementary Figure 9. Dynamic dopamine release in the dCA1 and dDG regions in response to environmental cues remained unchanged during the reinstatement phase.

(a) Schematic and representative images showing DA3h expression in dCA1. Scale bar, 400 μm. **(b, c)** Heatmaps and average traces demonstrate that dopamine dynamics in the dCA1 remained unchanged in response to DVCs during the reinstatement phase in cocaine-CPP-trained mice (b). Peak dopamine dynamics in the dCA1 showed no significant changes in response to DVCs during this phase (c). **(d)** Schematic and representative images showing DA3h expression in dDG. Scale bar, 400 μm. **(e, f)** Heatmaps and average traces demonstrate that dopamine dynamics in the dDG remained unchanged in response to DVCs during the reinstatement phase in cocaine-

CPP-trained mice (e). Peak dopamine dynamics in the dDG showed no significant changes in response to DVCs during this phase (f). Shaded areas around the means represent error bars. Color scales on the right indicate ΔF/F. dCA1, dorsal Cornu Ammonis 1; dCA3, dorsal Cornu Ammonis 3; dDG, dorsal Dentate Gyrus. All data are expressed as the mean ± s.e.m.

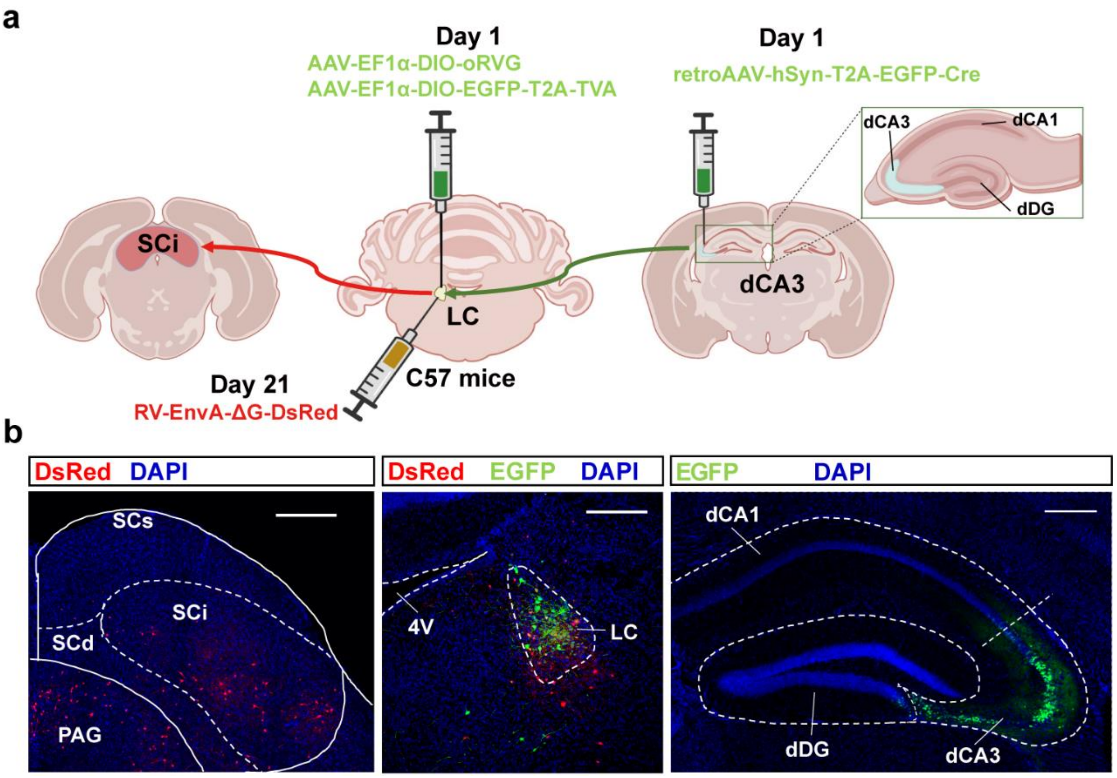

Supplementary Figure 10. Dissection of the SCi$^{CaMKII\alpha}$→LCTH→dCA3 Circuit.

(a) Schematic depicting viral injection for triple retrograde tracing. **(b)** Representative images showing viral expression in the indicated nuclei. Scale bars: SCi, 500 μm; LC, 200 μm; dCA3, 400 μm. SCs, superficial layers of the superior colliculus; SCi, intermediate layers of the superior colliculus; SCd, deep layers of the superior colliculus; PAG, periaqueductal gray; 4V, fourth ventricle; dCA1, dorsal Cornu Ammonis 1; dCA3, dorsal Cornu Ammonis 3; dDG, dorsal Dentate Gyrus.

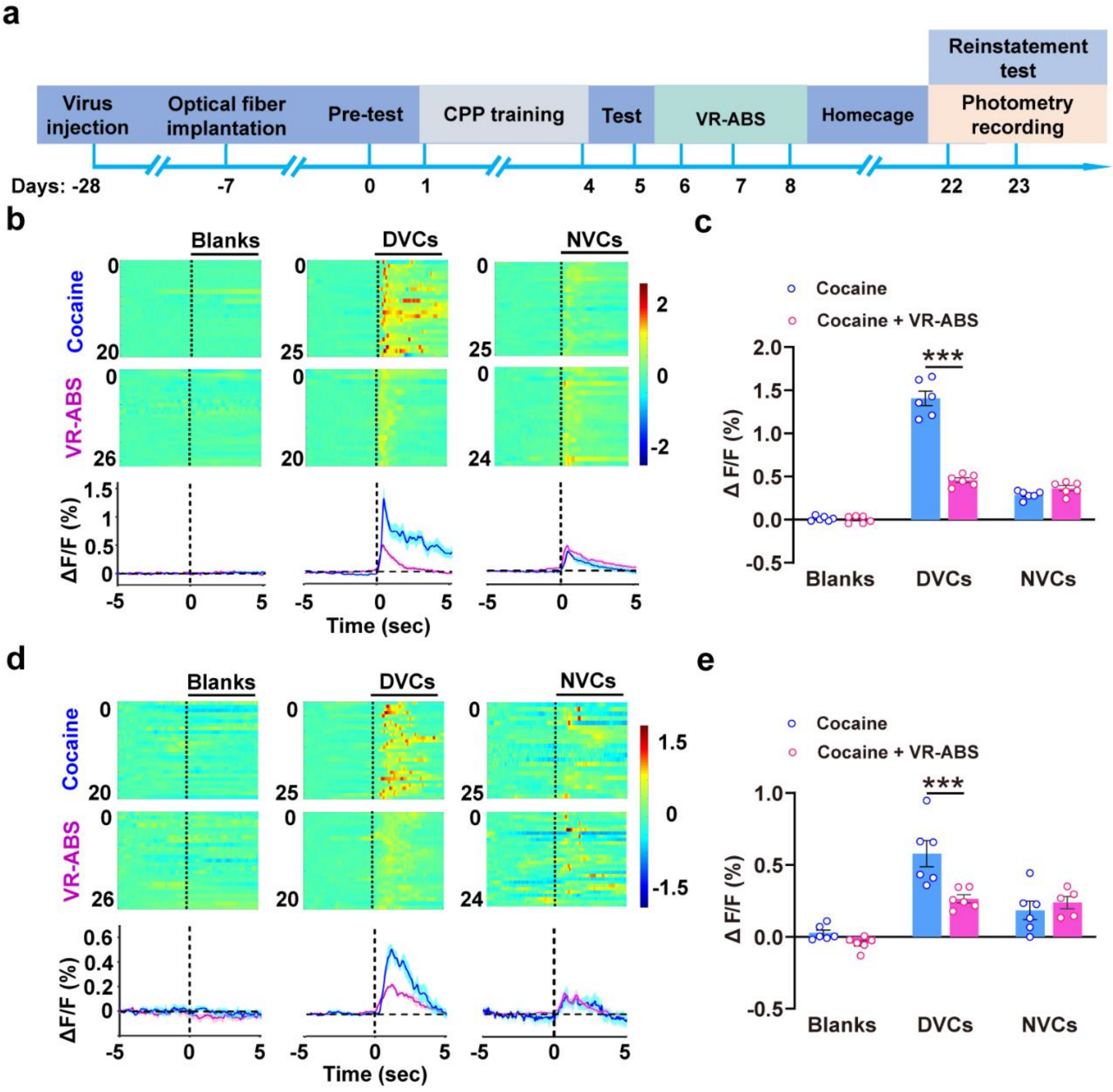

Supplementary Figure 11. VR-ABS treatment reverses the hyperactivation of LC^{TH} neurons and restores the increased dynamic release of dopamine in the dCA3 region in cocaine-CPP-trained mice.

(a) Experimental timeline illustrating in vivo photometry recordings following VR-ABS treatment. **(b)** Heatmaps and average calcium transients show that VR-ABS treatment reversed the increased activation of LC^{TH} neurons in response to DVCs during the reinstatement phase in cocaine-CPP-trained mice. **(c)** VR-ABS treatment reversed the elevated peak calcium transients in LC^{TH} neurons induced by DVCs during the reinstatement phase in cocaine-CPP-trained mice. **(d)** Heatmaps and average

dopamine transients show that VR-ABS treatment reversed the increased dopamine release within dCA3 neurons in response to DVCs during the reinstatement phase in cocaine-CPP-trained mice. **(e)** VR-ABS treatment reversed the elevated peak dopamine dynamics in dCA3 induced by DVCs during the reinstatement phase in cocaine-CPP-trained mice. Color scales on the right indicate ΔF/F values. All presented data are expressed as the mean ± s.e.m.; Statistical significance indicated as ***$p < 0.001$.

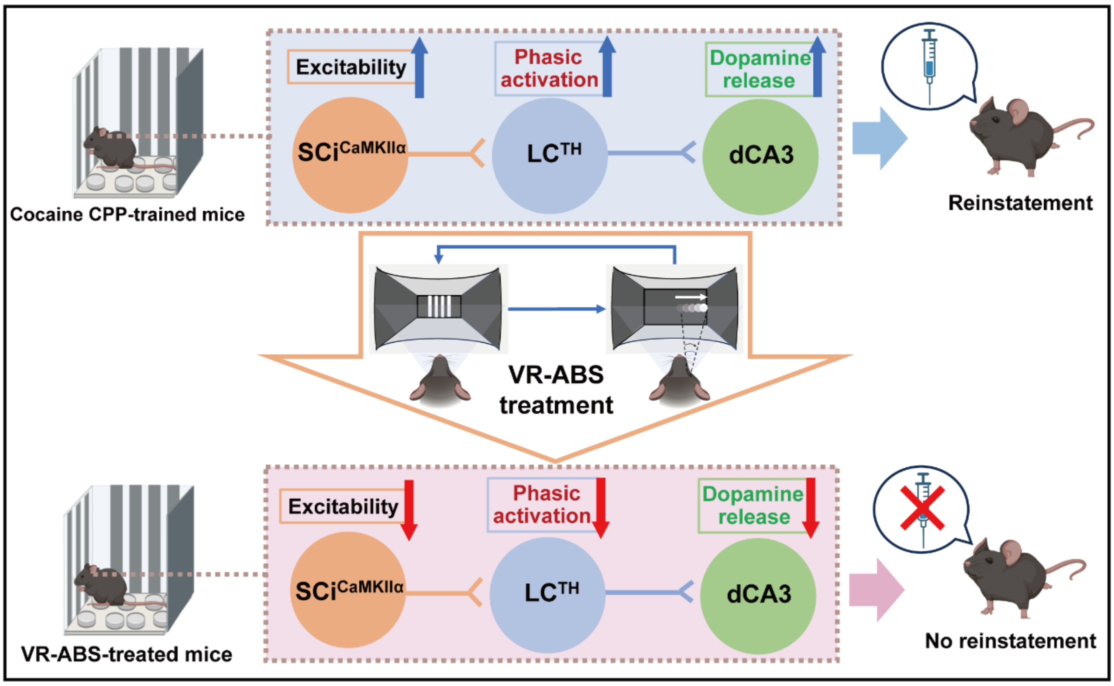

Supplementary Figure 12. VR-ABS prevents cocaine reinstatement by suppressing the SCi$^{CaMKII\alpha}\rightarrow$LC$^{TH}\rightarrow$dCA3 circuit.

Summary of the neural circuit mechanism involving the SCi$^{CaMKII\alpha}\rightarrow$LC$^{TH}\rightarrow$dCA3 pathway: During reinstatement, the excitability of SCi$^{CaMKII\alpha}$ neurons increases, making them more responsive to environmental visual cues. This heightened activity amplifies their output to LCTH neurons, resulting in enhanced phasic activity in LCTH neurons and increased dopamine release to dCA3, ultimately driving cocaine reinstatement. VR-ABS treatment reduces the excitability of SCi$^{CaMKII\alpha}$ neurons, thereby suppressing the activation of the SCi$^{CaMKII\alpha}\rightarrow$LC$^{TH}\rightarrow$dCA3 circuit and preventing cocaine reinstatement.

Supplementary Movie 1. Manipulating environmental visual stimuli via VR to induce cocaine reinstatement.

This movie demonstrates how environmental visual stimuli are manipulated within a virtual CPP testing environment using VR equipment. Mice are sequentially exposed to blank environments, DVCs, and NVCs, while their movement speed is concurrently tracked.

Supplementary Movie 2. VR-based stimulation treatments for cocaine reinstatement.

This movie illustrates the representative processes of VR extinction training and VR-ABS treatment. During VR extinction training, mice are exposed to DVCs. In VR-ABS treatment, after DVC exposure, mice receive visual stimulation with moving light spots to induce eye movement, aimed at facilitating extinction.

Supplementary Table 1. Virus strains.

REAGENT or RESOURCE	SOURCE	IDENTIFIER
AAV-CaMKIIα-GCaMP6s	BrainVTA Technology	Cat# PT-0110
AAV-CaMKIIα-hM4Di-mCherry	BrainVTA Technology	Cat# PT-0017
AAV-CaMKIIα-mCherry	BrainVTA Technology	Cat# PT-0108
AAV-EF1α-DIO-EGFP-T2A-TVA	BrainVTA Technology	Cat# PT-0062
AAV-EF1α-DIO-oRVG	BrainVTA Technology	Cat# PT-0023
RV-EnvA-ΔG-DsRed	BrainVTA Technology	Cat# R01002
AAV-EF1α-DIO-hM4Di-mCherry	BrainVTA Technology	Cat# PT4050
retroAAV-hSyn-T2A-Cre	OBiO Technology	Cat# CN-889
retroAAV-hSyn-T2A-EGFP-Cre	OBiO Technology	Cat# CN-890
AAV-EF1α-DIO-EGFP	BrainVTA Technology	Cat# PT-0795
AAV-CaMKIIα-ChR2-mCherry	BrainVTA Technology	Cat# PT-0005
AAV-CaMKIIα-NpHR-EGFP	BrainVTA Technology	Cat# PT-0008
AAV-EF1α-DIO-GCaMP6s	BrainVTA Technology	Cat# PT-0071
AAV-EF1α-DIO-ChR2-mCherry-	BrainVTA Technology	Cat# PT-0002
AAV-EF1α-DIO-mCherry	BrainVTA Technology	Cat# 0301719
AAV-EF1α-DIO-NpHR-EGFP	BrainVTA Technology	Cat# PT-0006
AAV-hSyn-DA3h	Brain Case	Cat# BC-0711
AAV-hSyn-NE2h	Brain Case	Cat# BC-0268
AAV-EF1α-DIO-hM4Di-mCherry	BrainVTA Technology	Cat# PT4050

Supplementary Table 2. Chemicals.

REAGENT or RESOURCE	SOURCE	IDENTIFIER
DAPI	Santa Cruz	Cat# sc-24941
Clozapine N-oxide dihydrochloride	MedChemExpress	Cat# HY-17366
Isoflurane	RWD Life Science	Cat# R510-22
Ascorbate acid	J&K Scientific	Cat# 171771
Choline chloride	Sigma	Cat# C7527
KCl	Sinopharm Chemical Reagent	Cat# 10016308
$CaCl_2$	Sinopharm Chemical Reagent	Cat# 10005817
$MgCl_2$	Sinopharm Chemical Reagent	Cat# XW77863031
$NaHCO_3$	Sinopharm Chemical Reagent	Cat# 10018960
NaH_2PO_4	Sinopharm Chemical Reagent limited corporation	Cat# 20040818
Glucose	Sinopharm Chemical Reagent limited corporation	Cat# 63005518
Mg_2SO_4	Sinopharm Chemical Reagent limited corporation	Cat# SM264301
Sucrose	Sinopharm Chemical Reagent limited corporation	Cat# 10021418
$ATP\text{-}Na_2$	Sigma	Cat# A26209
$GTP\text{-}Na_2$	Sigma	Cat# G8877
Creatine phosphate disodium salt tetrahydrate	J&K Scientific	Cat# 996463
CsCl	Sigma	Cat# 289329
Cesium methanesulfonate	Sigma	Cat# C1426
K-gluconate	Sigma	Cat# G4500
Potassium gluconate	Sigma	Cat# G4500
HEPES	Aladdin	Cat# H109407
Phosphocreatine	Sigma	Cat# P7936
EGTA	Sigma	Cat# E4378
QX314	Sigma	Cat# L5783
ATP-Mg	Sigma	Cat# A9187
Tetrodotoxin	Aladdin Biochemical Technology	Cat# 4368-28-9
4-aminopyridin	Sigma	Cat# 275875
D-AP5	MedChemExpress	Cat# HY-100714A
CNQX	Sigma	Cat# C239
Bicuculline	MedChemExpress	Cat# HY-N0219
SCH23390	Tocris	Cat# 0925
Sulpiride	Sigma	Cat# S8010
Propranolol	Sigma	Cat# P0884
Paraformaldehyde	Sinopharm Chemical Reagent	Cat# 80096618

Supplementary Table 3. Antibodies.

REAGENT or RESOURCE	SOURCE	IDENTIFIER
Rabbit anti-c-Fos	Abcam	Cat# 190289;<br>RRID: AB_2737414
Mouse anti-CaMKIIα	Cell Signaling Technology	Cat# 50049;<br>RRID: AB_2721906
Mouse anti-c-Fos	Santa Cruz	Cat# sc-166940;<br>RRID: AB_10609634
Rabbit anti-VGLUT1	Synaptic Systems	Cat# 135 302;<br>RRID: AB_2571617
Alexa Fluor 488 donkey anti-mouse	Invitrogen	Cat# A-21202;<br>RRID: AB_141607
Alexa Fluor 594 donkey anti-rabbit	Invitrogen	Cat# A-21207;<br>RRID: AB_141637
Alexa Fluor 488 donkey anti-rabbit	Invitrogen	Cat# A-21206<br>RRID: AB_2535792
Alexa Fluor 594 donkey anti-mouse	Invitrogen	Cat# A-21203<br>RRID: AB_141633

Supplementary Table 4. Statistical analysis for each figure.

**FIGURE NUMBER**	**TEST USED**	**n**	**P VALUE**	**DEGREES OF FREEDOM & F / t VALUE**

Fig. 1b	Two-way ANOVA followed by Bonferroni's multiple comparisons test	12, 12, 12, 12, 12, 12, 12, 12,	Interaction: p < 0.001<br>Row Factor: p < 0.001<br>Column Factor: p < 0.001<br>Saline - Cocaine<br>Pre-test: p > 0.99<br>Post-test: p < 0.001<br>Extinction test: p = 0.59<br>Reinstatement test: p < 0.001	F (3, 88) = 33.70<br>F (3, 88) = 25.32<br>F (1, 88) = 58.05
Fig. 1e	Two-way ANOVA followed by Bonferroni's multiple comparisons test	11, 10, 11, 10, 11, 10	Interaction: p < 0.001<br>Row Factor: p < 0.001<br>Column Facto: p = 0.0043<br>Cocaine - Saline<br>Blanks: p > 0.99<br>DVCs: p < 0.001<br>NVCs: p = 0.0695	F (2, 66) = 29.01<br>F (2, 66) = 44.61<br>F (1, 66) = 8.769
Fig. 1h	Two-way ANOVA followed by Bonferroni's multiple comparisons test	6, 7, 7, 6, 7, 7	Interaction: p < 0.001<br>Row Factor: p < 0.001<br>Column Factor: p = 0.14<br>Cocaine - Saline<br>Blanks: p > 0.99<br>DVCs: p < 0.001<br>NVCs: p = 0.09	F (2, 34) = 12.08<br>F (2, 34) = 80.08<br>F (1, 34) = 2.265
Fig. 1j	Two-way ANOVA followed by Bonferroni's multiple comparisons test	11, 13, 11, 13, 11, 13, 11, 13, 11, 13, 11, 13, 11, 13, 11, 13	Interaction: p = 0.0846<br>Row Factor: p < 0.001<br>Column Factor: p < 0.001<br>Saline - Cocaine<br>0: p > 0.99<br>50: p > 0.99<br>100: p > 0.99<br>150: p = 0.8722<br>200: p = 0.1756<br>250: p = 0.0404<br>300: p = 0.0133<br>350: p = 0.0035	F (7, 176) = 1.828<br>F (7, 176) = 35.66<br>F (1, 176) = 27.07
Fig. 1k	Unpaired t-test	11, 13	p = 0.006	t=3.025, df=22
Fig. 1l	Unpaired t-test	11, 13	p = 0.02	t=2.437, df=22
Fig. 1n right	Unpaired t-test	14, 14	p = 0.02	t=2.412, df=26
Fig. 1o right	Unpaired t-test	14, 14	p = 0.49	t=0.7005, df=26
Fig. 2c	Two-way ANOVA followed by Bonferroni's multiple comparisons test	8, 8, 8, 8, 8, 8, 8, 8, 8, 8, 8, 8, 8, 8, 8, 8, 8, 8, 8, 8, 8, 8,	Interaction: p < 0.001<br>Row Factor: p < 0.001<br>Column Facto: p < 0.001<br>Pre-test<br>Cocaine vs. Cocaine + Extinction: p > 0.99<br>Cocaine vs. Cocaine + VR extinction: p > 0.99<br>Cocaine vs. Cocaine + ABS: p > 0.99<br>Cocaine vs. Cocaine + VR-ABS: p > 0.99<br>Cocaine + Extinction vs. Cocaine + VR extinction: p > 0.99	F (12, 140) = 4.010<br>F (3, 140) = 110.7<br>F (4, 140) = 16.90

			Cocaine + Extinction vs. Cocaine + ABS: $p > 0.99$<br>Cocaine + Extinction vs. Cocaine + VR-ABS: $p > 0.99$<br>Cocaine + VR extinction vs. Cocaine + ABS: $p > 0.99$<br>Cocaine + VR extinction vs. Cocaine + VR-ABS: $p > 0.99$<br>Cocaine + ABS vs. Cocaine + VR-ABS: $p > 0.99$<br><br>CPP test<br>Cocaine vs. Cocaine + Extinction: $p > 0.99$<br>Cocaine vs. Cocaine + VR extinction: $p > 0.99$<br>Cocaine vs. Cocaine + ABS: $p > 0.99$<br>Cocaine vs. Cocaine + VR-ABS: $p > 0.99$<br>Cocaine + Extinction vs. Cocaine + VR extinction: $p > 0.99$<br>Cocaine + Extinction vs. Cocaine + ABS: $p > 0.99$<br>Cocaine + Extinction vs. Cocaine + VR-ABS: $p > 0.99$<br>Cocaine + VR extinction vs. Cocaine + ABS: $p > 0.99$<br>Cocaine + VR extinction vs. Cocaine + VR-ABS: $p > 0.99$<br>Cocaine + ABS vs. Cocaine + VR-ABS: $p > 0.99$<br><br>Extinction test<br>Cocaine vs. Cocaine + Extinction: $p = 0.06$<br>Cocaine vs. Cocaine + VR extinction: $p = 0.15$<br>Cocaine vs. Cocaine + ABS: $p = 0.49$<br>Cocaine vs. Cocaine + VR-ABS: $p < 0.001$<br>Cocaine + Extinction vs. Cocaine + VR extinction: $p > 0.99$<br>Cocaine + Extinction vs. Cocaine + ABS: $p > 0.99$<br>Cocaine + Extinction vs. Cocaine + VR-ABS: $p = 0.001$<br>Cocaine + VR extinction vs. Cocaine + ABS: $p > 0.99$<br>Cocaine + VR extinction vs. Cocaine + VR-ABS: $p < 0.001$<br>Cocaine + ABS vs. Cocaine + VR-ABS: $p < 0.001$<br><br>Reinstatement test<br>Cocaine vs. Cocaine + Extinction: $p > 0.99$<br>Cocaine vs. Cocaine + VR extinction:	

			p > 0.99<br>Cocaine vs. Cocaine + ABS: p > 0.99<br>Cocaine vs. Cocaine + VR-ABS: p < 0.001<br>Cocaine + Extinction vs. Cocaine + VR extinction: p > 0.99<br>Cocaine + Extinction vs. Cocaine + ABS: p > 0.99<br>Cocaine + Extinction vs. Cocaine + VR-ABS: p < 0.001<br>Cocaine + VR extinction vs. Cocaine + ABS: p = 0.35<br>Cocaine + VR extinction vs. Cocaine + VR-ABS: p < 0.001<br>Cocaine + ABS vs. Cocaine + VR-ABS: p < 0.001	
Fig. 2d	Two-way ANOVA followed by Bonferroni's multiple comparisons test	10, 10, 10, 10, 10, 10, 10, 10, 10, 10, 10, 10	Interaction: p < 0.001<br>Row Factor: p < 0.001<br>Column Facto: p < 0.001<br>Cocaine - Cocaine + VR-ABS<br>Blanks Test: p > 0.99<br>Blanks Relapse: p > 0.99<br>DVCs Test: p > 0.99<br>DVCs Relapse: p < 0.001<br>NVCs Test: p > 0.99<br>NVCs Relapse: p > 0.99	F (5, 108) = 19.56<br>F (5, 108) = 51.53<br>F (1, 108) = 15.37
Fig. 2f	Two-way ANOVA followed by Bonferroni's multiple comparisons test	6, 6, 6, 6, 7, 6	Interaction: p < 0.001<br>Row Factor: p < 0.001<br>Column Factor: p < 0.001<br>Cocaine - Cocaine + VR-AB<br>Blanks: p > 0.99<br>DVCs: p < 0.001<br>NVCs: p > 0.99	F (2, 31) = 41.73<br>F (2, 31) = 100.9<br>F (1, 31) = 33.75
Fig. 2h	Two-way ANOVA followed by Bonferroni's multiple comparisons test	14, 14, 14, 14, 14, 14, 14, 14, 14, 14, 14, 14, 14, 14, 14, 14, 14, 14, 14, 14, 14, 14, 14, 14	Interaction: p = 0.0756<br>Row Factor: p < 0.001<br>Column Factor: p < 0.001<br>0<br>Saline vs. Cocaine: p > 0.99<br>Saline vs. Cocaine + VR-ABS: p > 0.99<br>Cocaine vs. Cocaine + VR-ABS: p > 0.99<br>50<br>Saline vs. Cocaine: p = 0.7803<br>Saline vs. Cocaine + VR-ABS: p > 0.99<br>Cocaine vs. Cocaine + VR-ABS: p = 0.2820<br>100<br>Saline vs. Cocaine: p = 0.1080<br>Saline vs. Cocaine + VR-ABS: p > 0.99<br>Cocaine vs. Cocaine + VR-ABS: p = 0.0092<br>150<br>Saline vs. Cocaine: p = 0.0171	F (14, 312) = 1.608<br>F (7, 312) = 76.77<br>F (2, 312) = 46.06

			Saline vs. Cocaine + VR-ABS: p=0.9868<br>Cocaine vs. Cocaine + VR-ABS: p=0.0006<br>200<br>Saline vs. Cocaine: p = 0.0037<br>Saline vs. Cocaine + VR-ABS: p > 0.99<br>Cocaine vs. Cocaine + VR-ABS: p = 0.0002<br>250<br>Saline vs. Cocaine: p = 0.0014<br>Saline vs. Cocaine + VR-ABS: p > 0.99<br>Cocaine vs. Cocaine + VR-ABS: p < 0.001<br>300<br>Saline vs. Cocaine: p < 0.001<br>Saline vs. Cocaine + VR-ABS: p > 0.99<br>Cocaine vs. Cocaine + VR-ABS: p < 0.001<br>350<br>Saline vs. Cocaine: p = 0.001<br>Saline vs. Cocaine + VR-ABS: p > 0.99<br>Cocaine vs. Cocaine + VR-ABS: p = 0.0003	
Fig. 2i	One-way ANOVA followed by Bonferroni's multiple comparisons test	14, 14, 14	p = 0.67<br>Saline vs. Cocaine: p = 0.002<br>Saline vs. Cocaine + VR-ABS: p > 0.99<br>Cocaine vs. Cocaine + VR-ABS: p = 0.02	F (2, 39) = 7.487
Fig. 2j	One-way ANOVA followed by Bonferroni's multiple comparisons test	14, 14, 14	p = 0.8279<br>Saline vs. Cocaine: p = 0.0313<br>Saline vs. Cocaine + VR-ABS: p > 0.99<br>Cocaine vs. Cocaine + VR-ABS: p = 0.0111	F (2, 39) = 5.644
Fig. 2l right	One-way ANOVA followed by Bonferroni's multiple comparisons test	15, 15, 16	p = 0.02<br>Saline vs. Cocaine: p = 0.04<br>Saline vs. Cocaine + VR-ABS: p > 0.99<br>Cocaine vs. Cocaine + VR-ABS: p = 0.03	F (2, 43) = 4.539
Fig. 2m right	One-way ANOVA followed by Bonferron	15, 15, 16	p = 0.45<br>Saline vs. Cocaine: p = 0.73<br>Saline vs. Cocaine + VR-ABS: p > 0.99<br>Cocaine vs. Cocaine + VR-ABS: p =	F (2, 43) = 0.8057

	i's multiple comparisons test		0.97	
Fig. 3d	One-way ANOVA followed by Bonferroni's multiple comparisons test	10, 10, 10	Treatment (between columns): p < 0.001<br>ACSF vs. TTX + 4-AP: p > 0.99<br>ACSF vs. CNQX + D-AP5: p < 0.001<br>TTX + 4-AP vs. CNQX + D-AP5: p < 0.001	F (2, 27) = 17.60
Fig. 3f	Two-way ANOVA followed by Bonferroni's multiple comparisons test	8, 8, 8, 8, 8, 8, 8, 8, 8, 8	Interaction: p = 0.0087<br>Row Factor: p < 0.0001<br>Column Factor: p = 0.6338<br>EGFP - NpHR<br>Pre-test: p > 0.9999<br>Post-test: p = 0.3730<br>Extinction test: p > 0.9999<br>Off: p > 0.9999<br>On: p = 0.0063	F (4, 70) = 3.696<br>F (4, 70) = 30.25<br>F (1, 70) = 0.2290
Fig. 3g	Two-way ANOVA followed by Bonferroni's multiple comparisons test	8, 11, 8, 11,	Interaction: p = 0.0012<br>Row Factor: p = 0.0183<br>Column Factor: p < 0.001<br>Cocaine + NpHR - Cocaine + EYFP<br>Off: p = 0.3702<br>On: p < 0.001	F (1, 34) = 12.41<br>F (1, 34) = 6.144<br>F (1, 34) = 29.54
Fig. 3i	Two-way ANOVA followed by Bonferroni's multiple comparisons test	10, 10, 10, 10, 10, 10, 10, 10	Interaction: p = 0.005<br>Row Factor: p < 0.001<br>Column Factor: p = 0.03<br>ChR2 - mCherry<br>Pre-test: p > 0.99<br>Post-test: p > 0.99<br>Extinction test: p > 0.99<br>Off: p > 0.99<br>On: p < 0.001	F (4, 90) = 4.017<br>F (4, 90) = 15.43<br>F (1, 90) = 0.03
Fig. 3j	Two-way ANOVA followed by Bonferroni's multiple comparisons test	10, 10, 10, 10, 10, 10, 10, 10	Interaction: p = 0.07<br>Row Factor: p < 0.001<br>Column Factor: p = 0.23<br>Cocaine + VR-ABS + ChR2 - Cocaine + VR-ABS + mCherry<br>Pre-test: p > 0.99<br>CPP test: p > 0.99<br>Reinstatement Off: p > 0.99<br>Reinstatement On: p > 0.99	F (3, 72) = 2.446<br>F (3, 72) = 14.81<br>F (1, 72) = 1.452
Fig. 4c	Two-way ANOVA followed by Bonferroni's multiple comparisons test	6, 7, 7, 6, 7, 7	Interaction: p < 0.001<br>Row Factor: p < 0.001<br>Column Factor: p = 0.14<br>Cocaine – Saline<br>Blanks: p > 0.99<br>DVCs: p < 0.001<br>NVCs: p = 0 .09	F (2, 34) = 12.08<br>F (2, 34) = 80.08<br>F (1, 34) = 2.265
Fig. 4g	Unpaired t-test	9, 8	p = 0.013	t=3.960, df=15
Fig. 4i	Two-way	8, 8, 8,	Interaction: p < 0.0001	F (4, 70) = 7.191

	ANOVA followed by Bonferroni's multiple comparisons test	8, 8, 8, 8, 8, 8, 8	Row Factor: p < 0.0001<br>Column Factor: p = 0.2518<br>Cocaine + hM4Di - Cocaine + mCherry<br>Pre-test: p = 0.9603<br>Post-test: p > 0.9999<br>Extinction test: p > 0.9999<br>Saline: p > 0.9999<br>CNO: p < 0.0001	F (4, 70) = 28.81<br>F (1, 70) = 1.335
Fig. 4j	Two-way ANOVA followed by Bonferroni's multiple comparisons test	10, 10, 10, 10	Interaction: p = 0.0594<br>Row Factor: p = 0.0044<br>Column Factor: p = 0.0047<br>Cocaine + mCherry - Cocaine + hM4Di<br>Saline: p = 0.9111<br>CNO: p = 0.0025	F (1, 36) = 3.789<br>F (1, 36) = 9.251<br>F (1, 36) = 9.080
Fig. 5d	Two-way ANOVA followed by Bonferroni's multiple comparisons test	8, 8, 8, 8, 8, 8, 8, 8, 8, 8	Interaction: p = 0.0032<br>Row Factor: p < 0.0001<br>Column Factor: p = 0.0617<br>Cocaine + EYFP - Cocaine + NpHR<br>Pre-test: p > 0.9999<br>Post-test: p > 0.9999<br>Extinction test: p > 0.9999<br>Off: p > 0.9999<br>On: p = 0.0002	F (4, 70) = 4.394<br>F (4, 70) = 22.57<br>F (1, 70) = 3.605
Fig. 5e	Two-way ANOVA followed by Bonferroni's multiple comparisons test	10, 10, 10, 10	Interaction: p < 0.001<br>Row Factor: p < 0.001<br>Column Factor: p = 0.0045<br>Cocaine + NpHR - Cocaine + EGFP<br>Off: p = 0.2575<br>On: p < 0.001	F (1, 36) = 27.36<br>F (1, 36) = 26.14<br>F (1, 36) = 9.189
Fig. 5i	Two-way ANOVA followed by Bonferroni's multiple comparisons test	9, 9, 9, 9, 9, 9, 9, 9, 9, 9, 9, 9	Interaction: p = 0.002<br>Row Factor: p < 0.001<br>Column Factor: p = 0.07<br>ChR2 - mCherry<br>Pre-test: p > 0.99<br>Post-test: p > 0.99<br>Extinction test: p > 0.99<br>Off: p > 0.99<br>On 3-Hz: p > 0.99<br>On 12-Hz: p < 0.001	F (5, 96) = 4.269<br>F (5, 96) = 42.25<br>F (1, 96) = 3.243
Fig. 5j	Two-way ANOVA followed by Bonferroni's multiple comparisons test	10, 12, 10, 12, 10, 12, 10, 12, 10, 12	Interaction: p = 0.0507<br>Row Factor: p < 0.001<br>Column Factor: p = 0.0689<br>Cocaine + VR-ABS + ChR2 - Cocaine + VR-ABS + mCherry<br>Pre-test: p > 0.99<br>Test: p > 0.99<br>Reinstatement Off: p > 0.99<br>Reinstatement On - 3Hz: p > 0.99<br>Reinstatement On - 12Hz: p = 0.0024	F (4, 100) = 2.454<br>F (4, 100) = 32.16<br>F (1, 100) = 3.382
Fig. 6c	Two-way ANOVA followed	6, 6, 6, 6, 6, 6	Interaction: p = 0.01<br>Row Factor: p < 0.001<br>Column Factor: p = 0.001	F (2, 30) = 5.130<br>F (2, 30) = 37.66<br>F (1, 30) = 12.31

	by Bonferroni's multiple comparisons test		Cocaine -Saline<br>Blanks: p = 0.64<br>DVCs: p < 0.001<br>NVCs: p > 0.99	
Fig. 6g	Two-way ANOVA followed by Bonferroni's multiple comparisons test	7, 7, 7, 7, 7, 7, 7, 7	Interaction: p < 0.001<br>Row Factor: p < 0.001<br>Column Factor: p < 0.001<br>Cocaine + hM4Di - Cocaine + mCherry<br>Blanks Saline: p > 0.99<br>Blanks CNO: p > 0.99<br>DVCs Saline: p = 0.13<br>DVCs CNO: p < 0.001	F (3, 48) = 33.80<br>F (3, 48) = 139.9<br>F (1, 48) = 22.54
Fig. 6i	Two-way ANOVA followed by Bonferroni's multiple comparisons test	8, 8, 8, 8, 8, 8, 8, 8, 8, 8, 8, 8, 8, 8, 8, 8, 8, 8, 8, 8	Interaction: p < 0.001<br>Row Factor: p < 0.001<br>Column Factor: p = 0. 28<br>Pre-test<br>SCH23390 vs. ACSF: p > 0.99<br>Sulpiride vs. ACSF: p > 0.99<br>Propranolol vs. ACSF: p > 0.99<br>Sulpiride vs. SCH23390: p > 0.99<br>Propranolol vs. SCH23390: p > 0.99<br>Propranolol vs. Sulpiride: p > 0.99<br><br>Post-test<br>SCH23390 vs. ACSF: p > 0.99<br>Sulpiride vs. ACSF: p = 0.98<br>Propranolol vs. ACSF: p = 0.95<br>Sulpiride vs. SCH23390: p = 0.27<br>Propranolol vs. SCH23390: p = 0.26<br>Propranolol vs. Sulpiride: p > 0.99<br><br>Extinction test<br>SCH23390 vs. ACSF: p > 0.99<br>Sulpiride vs. ACSF: p > 0.99<br>Propranolol vs. ACSF: p > 0.99<br>Sulpiride vs. SCH23390: p > 0.99<br>Propranolol vs. SCH23390: p > 0.99<br>Propranolol vs. Sulpiride: p > 0.99<br><br>Test-1<br>SCH23390 vs. ACSF: p > 0.99<br>Sulpiride vs. ACSF: p > 0.99<br>Propranolol vs. ACSF: p > 0.99<br>Sulpiride vs. SCH23390: p > 0.99<br>Propranolol vs. SCH23390: p > 0.99<br>Propranolol vs. Sulpiride: p > 0.99<br><br>Test-2<br>SCH23390 vs. ACSF: p < 0.001<br>Sulpiride vs. ACSF: p = 0.40<br>Propranolol vs. ACSF: p > 0.99<br>Sulpiride vs. SCH23390: p < 0.001<br>Propranolol vs. SCH23390: p < 0.001<br>Propranolol vs. Sulpiride: p > 0.99	F (12, 140) = 4.120<br>F (4, 140) = 35.27<br>F (3, 140) = 1.290
Fig. 6j	Two-way	10, 10,	Interaction: p < 0.001	F (9, 143) = 9.009

	ANOVA followed by Bonferroni's multiple comparisons test	10, 10, 10, 10, 10, 10, 10, 10, 10, 9, 10, 10, 10, 10	Row Factor: p < 0.001<br>Column Factor: p < 0.001<br>ACSF<br>Blanks Tset-2 vs. Blanks Tset-1: p = 0.14<br>DVCs Tset-2 vs. DVCs Tset-1: p > 0.99<br>SCH23390<br>Blanks Tset-2 vs. Blanks Tset-1: p > 0.99<br>DVCs Tset-2 vs. DVCs Tset-1: p < 0.001<br>Propranolol<br>Blanks Tset-2 vs. Blanks Tset-1: p > 0.99<br>DVCs Tset-2 vs. DVCs Tset-1: p = 0.55<br>Sulpiride<br>Blanks Tset-2 vs. Blanks Tset-11: p > 0.99<br>DVCs Tset-2 vs. DVCs Tset-11: p > 0.99<br>DVCs Test-2<br>SCH23390 vs. ACSF: p < 0.001<br>Propranolol vs. ACSF: p > 0.99<br>Sulpiride vs. ACSF: p > 0.99<br>Propranolol vs. SCH23390: p < 0.001<br>Sulpiride vs. SCH23390: p < 0.001<br>Sulpiride vs. Propranolol: p > 0.99	F (3, 143) = 102.3<br>F (3, 143) = 21.41
Fig. S1a	Two-way ANOVA followed by Bonferroni's multiple comparisons test	6, 6, 6, 6, 6, 6, 6, 6	Interaction: p = 0.0013<br>Row Factor: p = 0.0002<br>Column Factor: p < 0.0001<br>Pre-test - Post-test<br>Saline: p > 0.9999<br>5 mg/kg cocaine: p = 0.5282<br>10 mg/kg cocaine: p = 0.0031<br>20 mg/kg cocaine: p < 0.001	F (3, 40) = 6.341<br>F (3, 40) = 8.307<br>F (1, 40) = 23.31
Fig. S1b	Two-way ANOVA followed by Bonferroni's multiple comparisons test	6, 6, 6, 6, 6, 6, 6, 6, 6, 6, 6, 6, 6, 6, 6, 6, 6, 6, 6, 6, 6, 6, 6, 6, 6, 6, 6, 6, 6, 6, 6, 6, 6, 6, 6, 6, 6, 6, 6, 6, 6, 6, 6, 6, 6, 6, 6, 6	Interaction: p < 0.0001<br>Row Factor: p < 0.0001<br>Column Factor: p < 0.0001<br>Sal vs. Coc 5mg: p < 0.001<br>Sal vs. Coc 10mg: p < 0.001<br>Sal vs. Coc 20mg: p < 0.001<br>Coc 5mg vs. Coc 10mg: p > 0.9999<br>Coc 5mg vs. Coc 20mg: p < 0.001<br>Coc 10mg vs. Coc 20mg: p < 0.001	F (33, 240) = 13.27<br>F (11, 240) = 15.99<br>F (3, 240) = 294.3
Fig. S1c	Two-way ANOVA followed by	6, 6, 6, 6, 6, 6, 6, 6	Interaction: p = 0.1484<br>Row Factor: p < 0.0001<br>Column Factor: p = 0.7878<br>Male mice + cocaine - Female mice +	F (3, 40) = 1.880<br>F (3, 40) = 71.73<br>F (1, 40) = 0.07340

	Bonferroni's multiple comparisons test		cocaine<br>Pre-test: p = 0.3716<br>DVCs: p > 0.9999<br>NVCs: p > 0.9999<br>Blanks: p > 0.9999	
Fig. S1e	Two-way ANOVA followed by Bonferroni's multiple comparisons test	6, 6, 6, 6, 6, 6, 6, 6, 6, 6	Interaction: p < 0.001<br>Row Factor: p < 0.001<br>Column Facto: p < 0.001<br>Cocaine - Saline<br>Pre-test: p > 0.99<br>Post-test: p < 0.001<br>Extinction test: p > 0.99<br>Tactile cues: p > 0.99<br>Visual cues: p < 0.001	F (4, 50) = 10.32<br>F (4, 50) = 9.941<br>F (1, 50) = 25.08
Fig. S1g	Two-way ANOVA followed by Bonferroni's multiple comparisons test	7, 7, 7, 7, 8, 8, 8, 8	Interaction: p = 0.0691<br>Row Factor: p < 0.001<br>Column Factor: p = 0.1325<br>Cocaine + control - Cocaine + blindfolds<br>Pre-test: p > 0.9999<br>Post-test: p > 0.9999<br>Extinction test: p > 0.9999<br>Reinstatement test: p = 0.0240	F (3, 52) = 2.507<br>F (3, 52) = 9.596<br>F (1, 52) = 2.335
Fig. S1i	Two-way ANOVA followed by Bonferroni's multiple comparisons test	6, 6, 6, 6, 6, 6, 6, 6, 6, 6	Interaction: p < 0.001<br>Row Factor: p < 0.001<br>Column Factor: p < 0.001<br>CPP - Control<br>Pre-test p > 0.99<br>Post-test: p < 0.001<br>Extinction test p > 0.99<br>NVCs p > 0.99<br>DVCs: p < 0.001	F (4, 50) = 13.20<br>F (4, 50) = 10.60<br>F (1, 50) = 36.45
Fig. S1k	Two-way ANOVA followed by Bonferroni's multiple comparisons test	6, 6, 6, 6, 6, 6, 6, 6, 6, 6	Interaction: p < 0.0001<br>Row Factor: p < 0.0001<br>Column Factor: p < 0.0001<br>Saline - Cocaine<br>Pre-test: p > 0.9999<br>Post-test: p = 0.1275<br>Extinction test: p < 0.0001<br>Localized DVCs: p < 0.0001<br>DVCs: p < 0.0001	F (4, 50) = 15.45<br>F (4, 50) = 11.89<br>F (1, 50) = 55.80
Fig. S2b SCs	Two-way ANOVA followed by Bonferroni's multiple comparisons test	3, 3, 3, 3, 3, 3	Interaction: p = 0.76<br>Row Factor: p = 0.06<br>Column Factor: P = 0.26<br>Control<br>DVCs vs. Blanks: p > 0.99<br>NVCs vs. Blanks: p = 0.75<br>NVCs vs. DVCs: p = 0.33<br>CPP<br>DVCs vs. Blanks: p = 0.43<br>NVCs vs. Blanks: p > 0.99<br>NVCs vs. DVCs: p = 0.18	F (2, 12) = 0.2846<br>F (2, 12) = 3.635<br>F (1, 12) = 1.386
Fig. S2b SCi	Two-way ANOVA followed by Bonferron	3, 3, 3, 3, 3, 3	Interaction: p = 0.05<br>Row Factor: p = 0.11<br>Column Factor: p = 0.06<br>CPP – Control<br>Blanks: p > 0.99	F (2, 12) = 3.730<br>F (2, 12) = 2.661<br>F (1, 12) = 4.217

	i's multiple comparisons test		DVCs: p = 0.02<br>NVCs: p > 0.99	
Fig. S2b SCd	Two-way ANOVA followed by Bonferroni's multiple comparisons test	3, 3, 3, 3, 3, 3	Interaction: p = 0.76<br>Row Factor: p = 0.51<br>Column Factor: p = 0.23<br>Control<br>DVCs vs. Blanks: p > 0.99<br>NVCs vs. Blanks: p > 0.99<br>NVCs vs. DVCs: p > 0.99<br>CPP<br>DVCs vs. Blanks: p = 0.85<br>NVCs vs. Blanks: p > 0.99<br>NVCs vs. DVCs: p > 0.99	F (2, 12) = 0.2382<br>F (2, 12) = 0.7064<br>F (1, 12) = 1.589
Fig. S3b	Two-way ANOVA followed by Bonferroni's multiple comparisons test	9, 9, 6, 6, 6, 7	Interaction: p = 0.01<br>Row Factor: p < 0.001<br>Column Factor: p = 0.01<br>CPP – Control<br>Blanks: p > 0.99<br>DVCs: p = 0.003<br>NVCs: p = 0.98	F (2, 37) = 4.854<br>F (2, 37) = 36.01<br>F (1, 37) = 6.572
Fig. S4c	Two-way ANOVA followed by Bonferroni's multiple comparisons test	8, 8, 8, 8, 8, 8, 8, 8, 8, 8	Interaction: p = 0.006<br>Row Factor: p < 0.001<br>Column Factor: p < 0.001<br>Cocaine + mCherry - Cocaine + hM4Di<br>Pre-test: p > 0.99<br>Post-test: p > 0.99<br>Extinction test: p = 0.12<br>Saline: p > 0.99<br>CNO: p < 0.001	F (4, 70) = 3.953<br>F (4, 70) = 44.14<br>F (1, 70) = 13.62
Fig. S4d	Two-way ANOVA followed by Bonferroni's multiple comparisons test	10, 7, 10, 7	Interaction: p < 0.001<br>Row Factor: p = 0.002<br>Column Factor: p < 0.001<br>mCherry - hM4Di<br>Saline: p = 0.11<br>CNO: p < 0.001	F (1, 30) = 15.48<br>F (1, 30) = 11.02<br>F (1, 30) = 46.09
Fig. S5e	Two-way ANOVA followed by Bonferroni's multiple comparisons test	6, 6, 6, 6, 6, 6,	Interaction: p < 0.001<br>Row Factor: p < 0.001<br>Column Factor: p < 0.001<br>Cocaine – Saline<br>Blanks: p > 0.99<br>DVCs: p = 0.001<br>NVCs: p > 0.99	F (2, 30) = 9.427<br>F (2, 30) = 45.85<br>F (1, 30) = 12.28
Fig. S5g	Two-way ANOVA followed by Bonferroni's multiple comparisons test	8, 8, 8, 8, 8, 8, 8, 8, 8, 8	Interaction: p = 0.04<br>Row Factor: p < 0.001<br>Column Factor: p = 0.04<br>Cocaine + hM4Di - Cocaine + mCherry<br>Pre-test: p > 0.99<br>Post-test: p > 0.99<br>Extinction test: p > 0.99<br>Saline: p > 0.99	F (4, 70) = 2.669<br>F (4, 70) = 38.97<br>F (1, 70) = 4.385

			CNO: p = 0.002	
Fig. S5h	Two-way ANOVA followed by Bonferroni's multiple comparisons test	9, 12, 9, 12	Interaction: p < 0.001<br>Row Factor: p = 0.10<br>Column Factor: p < 0.001<br>Cocaine + mCherry - Cocaine + hM4Di<br>Saline: p > 0.99<br>CNO: p < 0.001	F (1, 38) = 14.35<br>F (1, 38) = 2.830<br>F (1, 38) = 12.76
Fig. S6b	Two-way ANOVA followed by Bonferroni's multiple comparisons test	9, 9, 9, 9, 7, 9	Interaction: p = 0.07<br>Row Factor: p < 0.001<br>Column Factor: p = 0.003<br>Cocaine – Saline<br>Baseline: p = 0.37<br>DVCs: p = 0.002<br>NVCs: p > 0.99	F (2, 46) = 2.858<br>F (2, 46) = 41.28<br>F (1, 46) = 9.704
Fig. S6c	Two-way ANOVA followed by Bonferroni's multiple comparisons test	9, 9, 8, 9, 5, 9	Interaction: p = 0.12<br>Row Factor: p < 0.001<br>Column Factor: p = 0.04<br>Cocaine – Saline<br>Baseline: p = 0.43<br>DVCs: p = 0.03<br>NVCs: p > 0.99	F (2, 43) = 2.188<br>F (2, 43) = 11.59<br>F (1, 43) = 4.267
Fig. S6d	Two-way ANOVA followed by Bonferroni's multiple comparisons test	9, 9, 7, 8, 5, 8	Interaction: p = 0.17<br>Row Factor: p < 0.001<br>Column Factor: p = 0.11<br>Cocaine – Saline<br>Baseline: p > 0.99<br>DVCs: p = 0.05<br>NVCs: p > 0.99	F (2, 40) = 1.845<br>F (2, 40) = 17.19<br>F (1, 40) = 2.673
Fig. S6e	Two-way ANOVA followed by Bonferroni's multiple comparisons test	9, 9, 9, 9, 6, 9	Interaction: p = 0.09<br>Row Factor: p < 0.001<br>Column Factor: p = 0.02<br>Cocaine – Saline<br>Baseline: p > 0.99<br>DVCs: p = 0.005<br>NVCs: p > 0.99	F (2, 45) = 2.514<br>F (2, 45) = 22.56<br>F (1, 45) = 6.246
Fig. S7b	Two-way ANOVA followed by Bonferroni's multiple comparisons test	8, 8, 8, 8, 8, 8, 8, 8, 8, 8	Interaction: p = 0.2349<br>Row Factor: p < 0.0001<br>Column Factor: p = 0.4612<br>Cocaine + EYFP - Cocaine + NpHR<br>Pre-test: p = 0.1448<br>Post-test: p > 0.9999<br>Extinction test: p > 0.9999<br>Off: p > 0.9999<br>On: p > 0.9999	F (4, 70) = 1.425<br>F (4, 70) = 43.44<br>F (1, 70) = 0.5489
Fig. S7c	Two-way ANOVA followed by Bonferroni's multiple compariso	10, 10, 10, 10	Interaction: p = 0.3091<br>Row Factor: p = 0.4276<br>Column Factor: p = 0.0194<br>Cocaine + NpHR - Cocaine +EYFP<br>DVCs Off: p = 0.0376<br>DVCs On: p = 0.6468	F (1, 36) = 1.065<br>F (1, 36) = 0.6439<br>F (1, 36) = 5.992

	ns test			
Fig. S7e	Two-way ANOVA followed by Bonferroni's multiple comparisons test	8, 8, 8, 8, 8, 8, 8, 8, 8, 8	Interaction: p = 0.0365<br>Row Factor: p < 0.0001<br>Column Factor: p = 0.8833<br>Cocaine + EYFP - Cocaine + NpHR<br>Pre-test: p = 0.0505<br>Post-test: p > 0.9999<br>Extinction test: p = 0.5159<br>Off: p > 0.9999<br>On: p > 0.9999	F (4, 70) = 2.718<br>F (4, 70) = 49.76<br>F (1, 70) = 0.02169
Fig. S7f	Two-way ANOVA followed by Bonferroni's multiple comparisons test	10, 10, 10, 10	Interaction: p = 0.2655<br>Row Factor: p = 0.6844<br>Column Factor: p = 0.3847<br>Cocaine + NpHR - Cocaine +EYFP<br>Off: p = 0.3272<br>On: p > 0.99	F (1, 36) = 1.280<br>F (1, 36) = 0.1679<br>F (1, 36) = 0.7743
Fig. S8c	Two-way ANOVA followed by Bonferroni's multiple comparisons test	7, 6, 6, 6, 7, 7	Interaction: p = 0.53<br>Row Factor: p < 0.001<br>Column Factor: p = 0.95<br>Cocaine - Saline<br>Blanks: p > 0.99<br>DVCs: p > 0.99<br>NVCs: p > 0.99	F (2, 33) = 0.6538<br>F (2, 33) = 81.92<br>F (1, 33) = 0.004022
Fig. S8f	Two-way ANOVA followed by Bonferroni's multiple comparisons test	6, 6, 6, 6, 6, 6	Interaction: p = 0.07<br>Row Factor: p < 0.001<br>Column Factor: p = 0.73<br>Cocaine - Saline<br>Blanks: p > 0.99<br>DVCs: p = 0.24<br>NVCs: p = 0.38	F (2, 30) = 2.852<br>F (2, 30) = 37.50<br>F (1, 30) = 0.1176
Fig. S8i	Two-way ANOVA followed by Bonferroni's multiple comparisons test	6, 6, 6, 6, 6, 6	Interaction: p = 0.61<br>Row Factor: p < 0.001<br>Column Factor: p = 0.65<br>Cocaine - Saline<br>Blanks: p > 0.99<br>DVCs: p > 0.99<br>NVCs: p > 0.99	F (2, 30) = 0.4944<br>F (2, 30) = 10.18<br>F (1, 30) = 0.2141
Fig. S9c	Two-way ANOVA followed by Bonferroni's multiple comparisons test	5, 6, 6, 6, 6, 6	Interaction: p = 0.56<br>Row Factor: p < 0.001<br>Column Factor: p = 0.97<br>Cocaine - Saline<br>Blanks: p > 0.99<br>DVCs: p > 0.99<br>NVCs: p > 0.99	F (2, 29) = 0.5901<br>F (2, 29) = 25.11<br>F (1, 29) = 0.001362
Fig. S9f	Two-way ANOVA followed by Bonferroni's multiple comparisons	6, 6, 6, 6, 6, 7	Interaction: p = 0.25<br>Row Factor: p < 0.001<br>Column Factor: p = 0.55<br>Cocaine - Saline<br>Blanks: p > 0.99<br>DVCs: p > 0.99<br>NVCs: p = 0.27	F (2, 31) = 1.468<br>F (2, 31) = 19.59<br>F (1, 31) = 0.3577

	ns test			
Fig. S11c	Two-way ANOVA followed by Bonferroni's multiple comparisons test	6, 6, 6, 6, 6, 6	Interaction: $p < 0.001$<br>Row Factor: $p < 0.001$<br>Column Factor: $p < 0.001$<br>Cocaine + VR-ABS - Cocaine Blanks: $p > 0.99$<br>DVCs: $p < 0.001$<br>NVCs: $p = 0.26$	$F (2, 30) = 101.8$<br>$F (2, 30) = 277.9$<br>$F (1, 30) = 81.85$
Fig. S11e	Two-way ANOVA followed by Bonferroni's multiple comparisons test	6, 6, 6, 6, 6, 5	Interaction: $p = 0.005$<br>Row Factor: $p < 0.001$<br>Column Factor: $p = 0.01$<br>Cocaine + VR-ABS - Cocaine Blanks: $p > 0.99$<br>DVCs: $p < 0.001$<br>NVCs: $p > 0.99$	$F (2, 29) = 6.472$<br>$F (2, 29) = 35.58$<br>$F (1, 29) = 6.730$
